# Protease Inhibitors from Marine Venomous Animals and Their Counterparts in Terrestrial Venomous Animals

**DOI:** 10.3390/md11062069

**Published:** 2013-06-14

**Authors:** Caroline B.F. Mourão, Elisabeth F. Schwartz

**Affiliations:** Laboratory of Toxinology, Department of Physiological Sciences, University of Brasília, Brasília, DF, 70910-900, Brazil; E-Mail: carolcbfm@gmail.com

**Keywords:** protease inhibitor, Kunitz-type, serine protease, toxin, trypsin, chymotrypsin, venom

## Abstract

The Kunitz-type protease inhibitors are the best-characterized family of serine protease inhibitors, probably due to their abundance in several organisms. These inhibitors consist of a chain of ~60 amino acid residues stabilized by three disulfide bridges, and was first observed in the bovine pancreatic trypsin inhibitor (BPTI)-like protease inhibitors, which strongly inhibit trypsin and chymotrypsin. In this review we present the protease inhibitors (PIs) described to date from marine venomous animals, such as from sea anemone extracts and *Conus* venom, as well as their counterparts in terrestrial venomous animals, such as snakes, scorpions, spiders, Anurans, and Hymenopterans. More emphasis was given to the Kunitz-type inhibitors, once they are found in all these organisms. Their biological sources, specificity against different proteases, and other molecular blanks (being also K^+^ channel blockers) are presented, followed by their molecular diversity. Whereas sea anemone, snakes and other venomous animals present mainly Kunitz-type inhibitors, PIs from Anurans present the major variety in structure length and number of Cys residues, with at least six distinguishable classes. A representative alignment of PIs from these venomous animals shows that, despite eventual differences in Cys assignment, the key-residues for the protease inhibitory activity in all of them occupy similar positions in primary sequence. The key-residues for the K^+^ channel blocking activity was also compared.

## 1. Introduction

Protease inhibitors (PIs) are proteins or peptides capable of inhibiting the catalytic activity of proteolytic enzymes. They are widely distributed in nature and can be found in all kingdoms of cellular life and also in viral genomes [1,2]. A typical mammalian genome contains 2%–4% of genes encoding for proteases or protease inhibitors, reflecting the importance of proteolysis in their biological processes [3]. PIs have been known since the end of 20th century, and their identification can become more effective nowadays because of the current appliance of protease degradomics [4], which in association with proteomic tools and enzymatic assays can lead to the characterization of innumerous novel protease inhibitors.

Interest in characterizing new PIs and understanding their physiological significance has increased due to their biological relevance for all living processes, such as blood coagulation system, complement cascade, apoptosis, cell cycle and hormone processing pathways [5,6,7,8]. Furthermore, deficiencies or alterations in the regulation of these enzymes underlie several pathological conditions, such as cancer, arthritis, neurodegenerative and cardiovascular diseases [9,10].

According to the proteases they inhibit, PIs can be grouped primarily as serine, cysteine, aspartic and metallo protease inhibitors [11]. Among these, serine protease inhibitors are the largest and most widely distributed superfamily of PIs [1,12,13], and based upon their possession of conserved functional motifs they can be subdivided in many classes, being the Kunitz-type inhibitors the best characterized of them, probably due to their abundance in several organisms [2,14,15,16,17]. The Kunitz-type motif consists of a polypeptide chain of ~60 amino acid residues stabilized by three disulfide bridges (C_I_–C_VI_, C_II_–C_IV_, C_III_–C_V_). The Kunitz-type inhibitors interact with proteases by the classical substrate-like mechanism [13] mainly through the P1–S1 interaction, based on the nomenclature of Schechter and Berger [18]. The standard mechanism implies that substrates/inhibitors contain the reactive site residues P3, P2, P1, P1′, P2′, P3′, located in the most exposed region of the protease-binding loop, that bind to the substrate amino acid side chains S3, S2, S1, S1′, S2′, S3′, which form the groove or cleft where amide bond hydrolysis occurs. Non-prime and prime designations specify amino- and carboxy-terminal sides of cleavage site, respectively [11,18]. The Kunitz-type motif was first observed in the bovine pancreatic trypsin inhibitor (BPTI)-like protease inhibitors, which strongly inhibit serine proteases such as trypsin and chymotrypsin [19,20,21].

In this review we present the protease inhibitors described to date from marine venomous animals, most of which have been obtained from sea anemone extracts, but also from *Conus* species, as well as their counterparts in terrestrial venomous animals, such as snakes, scorpions, spiders, frogs and toads, and bees and wasps. Due to the big amount of data, we have given more emphasis on the Kunitz-type serine protease inhibitors, once they are the most studied compounds among PIs and are found in all these organisms. Initially, the PIs are presented according to their biological sources, together with their main characteristics and activities against different proteases. Then, their dual function including potassium channel blocking activity is discussed, followed by the molecular diversity of protease inhibitor compounds.

## 2. Protease Inhibitors from Sea Anemones

The first reports on the existence of protease inhibitors in sea anemones species date from the 70s [22,23]. Currently, protease inhibitor peptides and neurotoxins are isolated from sea anemone whole bodies, tentacles, secreted mucus and aggressive organs such as acrorhagi, which is present in some species from Actiniidae family [24]. 

Several PIs have already been isolated or partially purified and characterized from the sea anemone species *Actinia equina* [24,25,26], *Anemonia sulcata* [27,28,29,30], *Radianthus koseirensis* [31], *Heteractis crispa* (also named *Radianthus macrodactylus*) [32,33,34], *Rhodactis rhodostoma* [31], *Stoichactis* sp. [35], *Stoichactis helianthus* [36,37,38,39], *Stichodactyla haddoni* [40], *Anthopleura* aff. *xanthogrammica* [24,41], *Anthopleura elegantissima* [42] and *Anthopleura fuscoviridis* [24] (Table 1). Most of these characterized PIs are homologous to Kunitz-type inhibitors. However, some of them belong to different superfamilies.

**Table 1:** Protease inhibitors from venomous animals. Some protease inhibitors with less information about sequence or biological activity, as well as some putative protease inhibitors only found by means of transcriptomic approach but not tested against proteases, were not included in this table (some of them are found within the text, with the respective UniProtKB code). Organisms from which the PIs were obtained are indicated by the symbols at left: # sea anemones; + snakes; § scorpions; ¥ spiders; ¤ Anurans; ø Hymenopterans. Capital letters denote the proteases inhibited: T, trypsin; C, chymotrypsin; CL, cathepsin L; CB, cathepsin B; P, papain; K, kallikrein; PK, plasma kallikrein; TK, tissue kallikrein; Pl, plasmin; E, elastase; nE, neutrophil elastase; pE, pancreatic elastase; X, factor Xa; XII, α-factor XIIa; SA, subtilisin A; ptK, proteinase K; Th, thrombin. Structural classes are indicated by symbols: ♦ Kunitz-type motif protease inhibitors; ▯ Kazal-type protease inhibitors; ● thyroglobulin type-1domain; ◊ *Ascaris*-type motif; ○ Bowman-Birk-type motif.

Specie	Toxin	UniProt KB	AA ^a^	Protease inhibited	Inhibitory activity ^b–g^	References
# *Actinia equina*	♦ AEPI-I	-	59 ^III^	-	-	[26]
	♦ AEPI-II	-	59 ^III^	-	-	[26]
	● Equistatin	P81439	199 ^III^	Cathepsin L *,	0.051 (CL),	[25,43]
				papain,	0.57 (P),	
				cathepsin B	1.4 (CB) ^b^	
	♦ AEAPI	-	57 ^III^	Trypsin *, plasmin	700 IU/mg (T) ^c^	[24]
# *Anemonia sulcata*	■ Elastase inhibitor	P16895	48 ^III^	Porcine elastase	1.0 ^d^	[28,44]
	♦ Kalicludin-1 or AsKC1	Q9TWG0	58 ^III^	Trypsin	<30 ^d^	[27]
	♦ Kalicludin-2 or AsKC2	Q9TWF9	58 ^III^	Trypsin	<30 ^d^	[27]
	♦ Kalicludin-3 or AsKC3	Q9TWF8	59 ^III^	Trypsin	<30 ^d^	[27]
	♦ SA5 II	P10280	62 ^III^	Kallikrein, Trypsin	0.3 (T) ^d^	[29,32]
# *Anthopleura* aff. *xanthogrammica*	♦ AXPI-I or AXAPI	P81547	58 ^III^	Trypsin *, chymotrypsin, elastase, thermolysin	1900 IU/mg (T) ^c^	[24,41]
	♦ AXPI-II	P81548	58 ^III^	Trypsin *, chymotrypsin	490 IU/mg (T) ^c^	[41]
# *Anthopleura elegantissima*	♦ APEKTx1	P86862	65 ^III^	Trypsin	124 ^d^	[42]
# *Anthopleura fuscoviridis*	♦ AFAPI-I	-	56 ^III^	Trypsin *, plasmin	950 IU/mg (T) ^d^	[24]
	♦ AFAPI-III	-	56 ^III^	Trypsin *, plasmin	900 IU/mg (T) ^d^	[24]
# *Heteractis crispa*	♦ Jn-IV	P16344	56 ^III^	Trypsin	9.6 ^d^	[32,33]
	♦ InhVJ	-	56 ^III^	Trypsin *,	2.49 (T),	[45,46,47]
				chymotrypsin	21.7 (C) ^d^	
	♦ APHC1	B2G331	56 ^III^	Trypsin,	1000 (T),	[34]
				chymotrypsin	~5000 (C) ^d^	
# *Rhodactis rhodostoma*	♦ Inhibitor 4	-	48 ^II^	Trypsin, kallikrein *, chymotrypsin	0.95 (T), 0.49 (K), 33 (C) ^d^	[31]
# *Stichodactyla haddoni*	♦ SHTX-3	B1B5I8	62 ^III^	Trypsin	203 IU/mg ^c^	[40]
# *Stoichactis helianthus*	♦ ShPI-1	P31713	55^III^	Serine *,	0.11 (T),	[36,37]
				cysteine,	2.3 (C),	
				aspartic proteases	2.7 (Pl) ^d^	
	♦ ShPI-2	P81129	55 ^III^	Serine, cysteine, aspartic proteases	n.f.	[38]
+ *Dendroaspis polylepis polylepis*	♦ Dendrotoxin E	P00984	59 ^III^	Trypsin *,	1.0 (T),	[48]
				chymotrypsin	100 (C) ^d^	
+ *Daboia siamensis*	♦ BBPTI-1	-	60 ^III^	Chymotrypsin	4.77 ^b^	[49]
	♦ CBPTI-1	A8Y7N4	66 ^III^	Trypsin	407 ^b^	[50]
	♦ CBPTI-2	A8Y7N5	60 ^III^	Trypsin	666 ^b^	[50]
	♦ CBPTI-3	A8Y7N6	60 ^III^	Chymotrypsin	2.55 ^b^	[50]
+ *Hemachatus haemachatus*	♦ HHV inhibitor II	P00985	57 ^III^	Trypsin, chymotrypsin, kallikrein, plasmin	-	[51]
+ *Naja nivea*	♦ NNV inhibitor II	P00986	57 ^III^	Trypsin	-	[51]
+ *Vipera ammodytes*	♦ Trypsin inhibitor I	P00991	61 ^III^	Trypsin *, chymotrypsin, kallikrein, plasmin	0.34 (T), 270 (C) ^b^	[52,53]
	♦ Trypsin inhibitor II	-	62 ^III^	Trypsin *, chymotrypsin, kallikrein, plasmin	0.56 (T), 300 (C) ^b^	[52]
	♦ Chymotrypsin inhibitor	P00992	65 ^III^	Chymotrypsin *, trypsin, plasma kallikrein	4.3 (C), 5100 (T) ^b^	[52]
+ *Bungarus fasciatus*	♦ BF9	P25660	65 ^III^	Chymotrypsin	58 ^d^	[54]
	♦ Bungaruskunin	B2KTG1	59 ^III^	Chymotrypsin *, trypsin, elastase	6100 (C) ^b^	[55]
+ *Naja naja*	♦ Trypsin inhibitor	P20229	57 ^III^	Trypsin	0.0035 ^b^	[56]
+ *Naja atra*	♦ NACI	Q5ZPJ7	57 ^III^	Chymotrypsin	25 ^b^	[57,58]
+ *Ophiophagus hannah*	♦ Oh11-1	P82966	58 ^III^	Chymotrypsin	3520 ^b^	[59]
	♦ OH-TCI	B6RLX2	58 ^III^	Chymotrypsin *,	84.6 (C),	[60]
				trypsin	391 (T) ^b^	
+ *Bungarus multicinctus*	♦ PILP-1	B4ESA2	58 ^III^	Trypsin	55.62 ^b^	[61]
+ *Oxyuranus scutellatus scutellatus*	♦ TSPI	B7S4N9	62 ^III^	Kallikrein *, trypsin, plasmin, elastase, factor Xa, α-factor XIIa	0.057 (PK), 0.23 (TK), 0.31 (T), 6.1 (Pl), 201 (E), 871 (X), 2380 (XII) ^b^	[62,63]
+ *Pseudonaja textilis textilis*	♦ Textilinin-1	Q90WA1	59 ^III^	Plasmin *,	0.49 ± 0.02 (Pl),	[7,63]
				trypsin	0.76 ± 0.02 (T) ^b^	
	♦ Textilinin-2	Q90WA0	59 ^III^	Plasmin	2.2 ^b^	[7]
+ *Macrovipera lebetina transmediterranea*	♦ PIVL	I2G9B4	67 ^III^	Trypsin	-	[64]
+ *Pseudechis australis*	♦ *Pr*-mulgin 1	E7FL11	59 ^III^	Metalloprotease 2	60 ^b^	[65]
	♦ *Pr*-mulgin 2	E7FL12	59 ^III^	Trypsin *,	5 (T),	[65]
				chymotrypsin,	40 (C),	
				plasmin	40 (Pl) ^b^	
	♦ *Pr*-mulgin 3	E7FL13	59 ^III^	Trypsin,	5 (T),	[65]
				plasmin	100 (Pl) ^b^	
§ *Mesobuthus tamulus*	♦ Fraction IX-1-a	-	-	Trypsin *,	19.2 IU/mg (T),	[66]
				kallikrein	87 IU/mg (K) ^c^	
§ *Hadrurus gertschi*	♦ rHg1	P0C8W3	67 ^III^	Trypsin	107 ^d^	[67,68]
§ *Lychas mucronatus*	♦ rLmKTT-1a	P0DJ46	59 ^III^	Trypsin	140 ^d^	[16,68]
	♦ rLmKTT-1b or SdPI	P0DJ45	59 ^III^	Trypsin	160 ^d^	[16,68]
	♦ rLmKTT-1c	P0DJ48	59 ^III^	Trypsin	124 ^d^	[68]
§ *Mesobuthus martensii*	♦ rBmKTT-1	P0DJ49	59 ^III^	Trypsin	136 ^d^	[68]
	♦ rBmKTT-2	P0DJ50	58 ^IV^	Trypsin	420 ^d^	[68]
	♦ rBmKTT-3	P0DJ47	70 ^III^	Trypsin	760 ^d^	[68]
§ *Scorpiops jendeki*	◊ rSjAPI	-	64 ^V^	Chymotrypsin *,	97.1 (C),	[69]
				elastase	3700 (E) ^b^	
¥ *Ornithoctonus huwena*	♦ HWTX-XI	P68425	55 ^III^	Trypsin	0.23 ^d^	[14]
¥ *Araneus ventricosus*	♦ AvKTI	K7YYJ2	57 ^III^	Trypsin,	7.34 (T),	[70]
				chymotrypsin,	37.75 (C),	
				plasmin *,	4.89 (Pl),	
				neutrophil elastase	169.07 (E) ^b^	
	♦ AvCI	L7X735	70 ^IV^	Chymotrypsin,	49.85 (C),	[71]
				subtilisin A,	20.51 (SA),	
				proteinase K,	65.42 (ptK),	
				neutrophil elastase,	8.74 (nE),	
				pancreatic elastase	11.32 (pE) ^b^	
¤ *Bombina bombina*	◊ BSTI	Q90248	60 ^V^	Trypsin,	80–100 (T),	[72]
				thrombin	1300 (Th) ^b^	
¤ *Bombina maxima*	◊ BMTI	Q8QFP3	60 ^V^	Trypsin	60 ^b^	[73]
¤ *Rana areolata*	◊ Trypsin inhibitor	-	61 ^V^	Trypsin	~20,000 ^e^	[74]
¤ *Bombina orientalis*	◊ BOTI	Q800F0	60 ^V^	-	-	[75]
¤ *Bombina variegata*	◊ BVTI	Q800E9	60 ^V^	-	-	[75]
¤ *Bombina microdeladigitora*	◊ BMSI 1	B1P2F8	60 ^V^	Trypsin, thrombin	20 (T), 150 (Th) ^b^	[76]
	◊ BMSI 2	B1P2F9	60 ^V^	-	-	[76]
¤ *Hyla simplex*	Hylaserpin S1	H6SWK9	392	Trypsin,	55 (T),	[77]
				chymotrypsin	310 (C) ^b^	
	◊ Hylaserpin S2	H6SWL0	56 ^V^	Trypsin	72 ^b^	[77]
¤ *Bufo andrewsi*	BATI	-	-	Trypsin	14 ^b^	[78]
				Trypsin	4.6 × 10^6^ (T),	
	Baserpin	-	-	chymotrypsin,	8.9 × 10^6^ (C),	[79]
				elastase	6.8 × 10^6^ (E) ^f^	
¤ *Kaloula pulchra hainana*	KPHTI	-	-	Trypsin	27 ^c^	[80]
¤ *Phyllomedusa sauvagii*	■ PSKP-1	P83578	58 ^III^	Prolyl endopeptidase	124 ± 56 ^g^	[81]
	■ PSKP-2	P83579	58 ^III^	-	-	[81]
¤ *Phyllomedusa nordestina*	PI01	K9N0E2	78 ^III^	-	-	[82]
	PI02	K9N1K5	77 ^III^	-	-	[82]
	PI03	K9N2T9	53 ^III^	-	-	[82]
¤ *Agalychnis callidryas*	■ ACKTI	I7J523	52 ^III^	Trypsin	1.9 ^b^	[83]
¤ *Dyscophus guineti*	♦ Kunitz-type PI	J9UVV9	57 ^III^	Trypsin	-	[17]
¤ *Kassina senegalensis*	♦ KSCI	F8K9Q3	62 ^III^	Chymotrypsin	-	[84]
¤ *Hyla annectans*	♦ Anntoxin	C7AR58	60 ^II^	Trypsin	25 ^b^	[85]
¤ *Odorrana grahami*	OGTI	-	17 ^II^	Trypsin	400 ^b^	[86]
¤ *Huia versabilis*	HV-BBI	B1VC43	18 ^I^	Trypsin	18.8 + 1.8 ^b^	[87]
ø *Vespa bicolor* Fabricius	♦ Bicolin	C0LNR2	54 ^III^	Trypsin *, thrombin	550 (T), 26,000 (Th) ^b^	[88]
ø *Bombus ignitus*	♦ Bi-KTI	G3LH89	58 ^III^	Plasmin	43.53 ^g^, 3.6 ^b^	[6]
ø *Bombus terrestris*	♦ Bt-KTI	D8KY58	58 ^III^	Plasmin	2.01 ^b^	[89]

* Protease against which the inhibitor showed more potent activity; ^a^ Number of amino acid residues and disulfide bridges (superscript) of the mature peptide; ^b^ Measured in terms of *K*_i_ value (nM); ^c^ Measured in terms of inhibitory units (IU)/mg, where 1 IU is the amount of protein that inhibit one unit of enzyme; ^d^ Measured in terms of *K*_d_ value (nM); ^e^ Unique concentration used (nM); ^f^ Association rate (*k*_ass_) measured in M^−1^·s^−1^; ^g^ Measured in terms of IC_50_ value (nM); n.f. means inhibitory activity not found by the authors; - Means data not available in the literature.

Equistatin, a protease inhibitor isolated from the hydrophilic extract of the whole body of *Actinia equina* [25], is an acidic protein composed of three thyroglobulin type-1 domains [43]. It is encoded by a putative sequence of 231 amino acids, including the signal peptide [43]. Equistatin inhibits papain-like cysteine proteases, such as papain and cathepsin L, with lower affinity for cathepsin B (Table 1) [25]. It was further shown that the *N*-terminal domain alone acts as a Cys protease inhibitor (*K*_i_ of 0.6 nM for papain), and the second domain act as an aspartic protease inhibitor (*K*_i_ of 0.3 for cathepsin D). The function of the third domains remains unknown [90,91].

From the extracts of *Anemonia sulcata* it was isolated an elastase inhibitor (AEI) that was found to be a non-classical Kazal-type inhibitor with respect to positioning of the cysteine residues [28,30,44]. With 48 amino acid residues, its tridimensional structure resembles those of typical Kazal-type inhibitors, however, the disulfide bridge C_I_–C_V_ in the sea anemone elastase inhibitor is shifted by one turn in the α-helical segment towards the *C*-terminal in comparison with those of Kazal-type’s [28,30]. The inhibitor strongly inhibits porcine pancreatic elastase (Table 1), with weaker activity against human leucocytes elastase (*K*_i_ of 1.0 µM). No inhibition was observed against other serine proteases such as bovine trypsin, bovine chymotrypsin, subtilisin from *Bacillus subtilis* and cathepsin G from human leucocytes [44].

Type II toxins from sea anemone are a peptide group that block K_v_1 channel currents—although with much less potency than the sea anemone type I toxins, which are potent K_v_1 channel blockers [92]—and are characterized by a polypeptide chain of 58–63 amino acid residues and three disulfide bridges [42,93]. They are homologous to Kunitz-type inhibitors of serine proteases and their biological role is still unclear. It is supposed that these protease inhibitors could (1) defend sea anemones from the protease of their victims; (2) protect the toxins injected into preys or predators from fast degradation; (3) act on the regulation of digestive mechanisms, including self-digestion by their own enzymes or by those of symbiotic microorganism; (4) and also, due to their dual activity, they could also be used to paralyze preys [31,35,92].

Among these Kunitz-type inhibitor toxins (KTTs), the sea anemone kalicludines (AsKC1 to AsKC3, Table 1), from *Anemonia sulcata*, are examples of toxins with both protease inhibitor and potassium channel blocking activities (Table 2) [27]. With 58–59 amino acid residues cross-linked by three disulfide bridges, all the three kalicludines inhibit trypsin with very similar inhibition profiles, in a 1:1 molar ratio, such as BPTI. From titration curves *K*_d_ values below 30 nM could be deduced [27]. Another KTT from *A. sulcata* is the Kunitz-type protease inhibitor 5 II (SA5 II, Table 1), which inhibits both tissue and plasma kallikreins [29]. Previous to these studies, at least ten KTTs with inhibitory effects against trypsin, chymotrypsin, plasmin and kallikrein have been reported from *A. sulcata* extracts [22,23].

**Table 2:** Potassium channel blocking activity of Kunitz-type motif protease inhibitor peptides from animal venoms. Scorpion and spider mutated peptides were not considered.

Source	Peptide	IC_50_ (nM)	Target	Ref.
Sea anemone	APEKTx1 *	0.9 ± 0.1	K_v_1.1	[42]
	AsKC1 *	2800	K_v_1.2	[27]
	AsKC2 *	1100	K_v_1.2	[27]
	AsKC3 *	1300	K_v_1.2	[27]
	SHTX-3 *	650	Synap ^a^	[40]
Cone snail	Conk-S1	1.33 ± 0.5	*Shaker*	[94]
Snake	α-DTX	0.4–150	K_v_1.1, K_v_1.2, K_v_1.6	[95]
	DTX-I	0.13–50	K_v_1.1, K_v_1.2, K_v_1.6	[95]
	DTX-K	0.03	K_v_1.1	[96]
	δ-DTX	0.029–1.8	K_v_1.1	[96,97]
	DaE	300	K_v_1.1	[98]
Scorpion	Hg1 *	6.2 ± 1.2	K_v_1.3	[68]
	LmKTT-1a *	1580	K_v_1.3	[68,99]
	LmKTT-1b *	>1000	K_v_1.3	[68]
	LmKTT-1c *	>1000	K_v_1.3	[68]
	BmKTT-1 *	129.7 ± 31.3	K_v_1.3	[68]
	BmKTT-2 *	371.3 ± 82.1	K_v_1.3	[68]
	BmKTT-3 *	>1000	K_v_1.3	[68]
Spider	HWTX-XI *	2570	K_v_1.1	[14]

* Peptides that also present protease inhibitory activity, as shown in Table 1; ^a^ Activity obtained through synaptosomal binding assay.

In the 80s, three protease inhibitors composed of 52–56 amino acid residues were isolated from *Stoichactis* sp. [35]. Inhibitors 2 and 3 had estimated molecular masses of ~6000 Da. Inhibitor 2, which was the main component, had its approximate molecular mass (5800 Da) determined by gel filtration. Inhibitors 2 and 3 lack tryptophan in their structures and contain two and three disulfide bridges, respectively. They both inhibited trypsin almost completely (98%) and, to a lesser extent, also chymotrypsin (86% and 92%, respectively). Inhibitor 1, which was still impure, presented a lower inhibitory activity (80% against trypsin and 50% against chymotrypsin). 

In the same decade, protease inhibitors were isolated from the body extracts of *Radianthus koseirensis* and *Rhodactis rhodostoma* [31]. From *R. rhodostoma*, the main protease inhibitor has 5459 Da and only two disulfide bridges. Named inhibitor 4 (Table 1), it had a high affinity for serine proteases (trypsin, kallikrein and chymotrypsin), and was less active in terms of the molar ratio (enzyme:inhibitor). Whereas typical trypsin inhibitors act stoichiometrically 1:1, inhibitor 4 from *R. rhodostoma* had a ratio of 1:2 for trypsin and 1:2.4 for chymotrypsin and kallikrein. From *R. koseirensis*, an inhibitor fraction was obtained, but it had very low affinity for serine proteases and did not inhibit chymotrypsin. The authors suggest that the ineffectiveness towards serine proteases of *R. koseirensis* fraction may have its origin in the neutral to slightly acidic properties of its inhibitor molecule. Differently, PIs from *R. rhodostoma* are basic in nature, such as BPTI and many KTTs [31].

**Figure 1:**
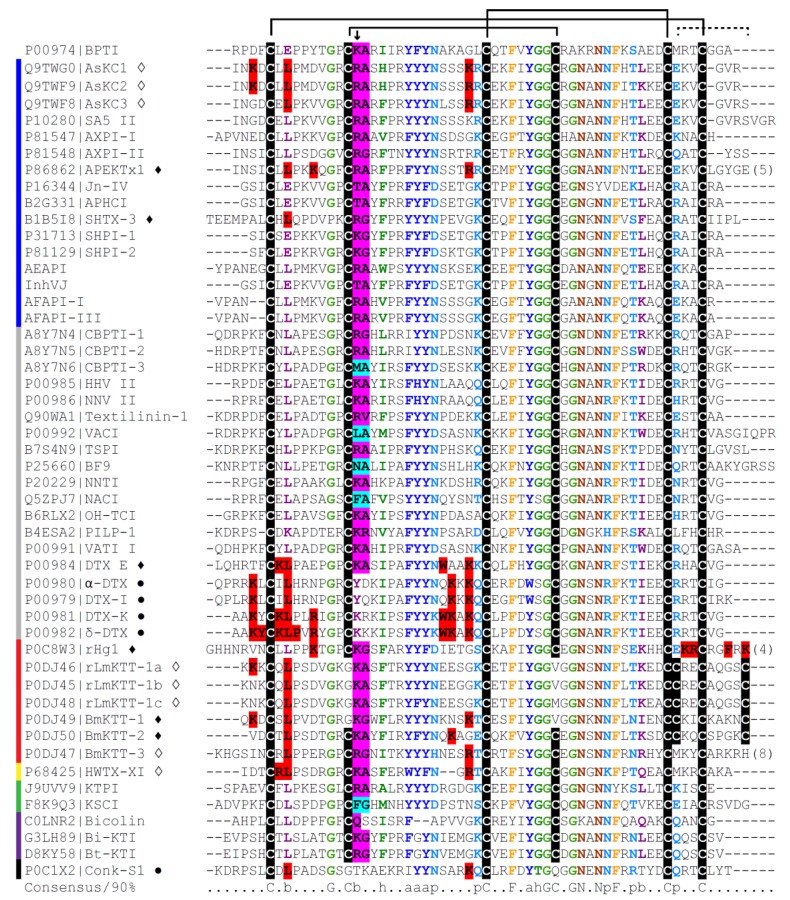
Representative alignment of toxins with the Kunitz-type motif from venomous animals. Organisms from which the polypeptides were obtained are indicated by the colored lines at left: blue, sea anemones; grey, snakes; red, scorpions; yellow, spider; green, Anurans; purple, Hymenopterans; black, cone snail. The toxins that present potassium channel blocking activity are indicated by symbols after their names: ●, K^+^ channel blockers with no protease inhibitory activity or still not tested against proteases; ♦ and ◊, dual-function toxins, where ♦ denotes toxins with stronger potency in K^+^ channels than those indicated by ◊. The other polypeptides, without symbols, are all serine protease inhibitors. The alignment was generated by ClustalW [170] and the consensus sequence was colored using Chroma and manual edition [171]. Key residues for K^+^ channel blocking activity are highlighted in red. Key residues for protease inhibition with more specificity to trypsin or chymotrypsin are highlighted in magenta and cyan, respectively. Some of them were suggested to be key residues by sequence similarity with other toxins. The P1 site residues are pointed by an arrow. Capital letters denote amino acids. Lower-case letters denote: b, big; p, polar; h, hydrophobic; a, aromatic. The known conserved disulfide bridges are labeled in black lines. The black dotted line suggests a possible new disulfide bridge in scorpion venom PIs [99]. The numbers within parenthesis mean amino acid residues from the *C*-terminus of the peptides that were not completely shown in this alignment.

Until now, many PIs were isolated from the body extract of *Heteractis crispa* (=*Radianthus macrodactylus*), but only few of them were fully characterized. Protease inhibitors of about 6000 Da were obtained from a water-ethanol extract of *H. crispa*, and the primary structure was elucidated for one of them, named Kunitz-type trypsin inhibitor IV or Jn-IV (Table 1) [33]. After that, four trypsin inhibitors were isolated (InI–InIV), and one of them (InI) was partially characterized. Composed by 59 amino acid residues, its molecular mass determined by SDS-PAGE was 7100 Da, whereas its molecular mass calculated by the amino acid composition was 6304 Da [100]. Posteriorly, two serine protease inhibitors were isolated [32]. RmIn I and RmIn II have 6322.8 and 6096.0 Da, respectively, according to their amino acid composition, and both have three disulfide bridges and no methionine or tryptophan residues [32], which is common in Kunitz-type protease inhibitors [35,51,101], with few exceptions (Figure 1). The *N*-terminal amino acid sequences of RmIn I and RmIn II were determined and they were 66%–86% identical with the analogous fragment of Kunitz-type trypsin inhibitor IV, isolated from the same source, suggesting that they are isoforms. Both RmIn I and RmIn II were active against trypsin and α-chymotrypsin, with *K*_i_ values of 2.4 and 2.5 nM for trypsin and 23 and 30 nM for α-chymotrypsin, respectively. Moreover, both polypeptides were not toxic to mice (10–100 µg of peptide per kg of animal) and exhibited antihistamine activity in a dose-dependent manner [32].

Also from *Heteractis crispa* extract, a Kunitz-type toxin designated as InhVJ was isolated (Table 1) [45,46]. It has 6106 Da, three disulfide bridges and also no Met or Trp residues [45,47]. Its putative sequence encodes a 78-amino acid residues polypeptide, including a signal peptide with 22 amino acid residues [47]. InhVJ is highly specific toward trypsin and α-chymotrypsin and did not inhibit other serine proteases (such as thrombin, kallikrein and plasmin), neither cysteine (papain) nor aspartic (pepsin) proteases [45,46]. Recently characterized, the analgesic polypeptide HC1 (or APHC1), with 6187.0 Da, presents very weak inhibition against trypsin and chymotrypsin (Table 1), although it completely inhibits both at a 1:1 molar ratio. Its main activity is related to an analgesic effect due to modulation of mammalian TRPV1-receptors [34].

The peptides ShPI-1 and ShPI-2 were obtained from the crude extract of *Stoichactis helianthus*. With 6110.6 Da and six cysteine residues, ShPI-1 was active against many proteases of different mechanistic classes [37]. It inhibited serine, cysteine and aspartic proteases, and did not affect the one metalloenzyme tested (bacterial collagenase). The strongest inhibition capacity was against trypsin (*K*_i_ = 1.1 × 10^−10^ M). Other serine proteases such as plasmin and chymotrypsin, and cysteine proteases (bromelain and papain) were inhibited with smaller intensity. The smallest degrees of inhibition were obtained against the serine protease kallikrein and elastase and against aspartic proteases [37]. 

Four serine protease inhibitors (AEPI-I to -IV) were isolated from the sea anemone *Actinia equina* [26]. Whereas only 35 and 37 *N*-terminal amino acid residues were determined for AEPI-III and -IV, the complete primary sequence was determined to AEPI-I and -II, which contain 59 amino acid residues and three disulfide bonds. Both peptides (Table 1) share structural similarity with Kunitz-type inhibitors, however, their protease inhibitory activity has not been measured yet.

From the whole body extract of *Stichodactyla haddoni*, four peptide toxins were isolated and named SHTX-1 to SHTX-4 [40]. SHTX-1–3 induced paralysis in crabs with ED_50_ values of 430, 430 and 183 µg/kg, respectively, exhibiting no lethality even at 1000 µg/kg. SHTX-4 was lethal to crab with an estimated LD_50_ of 93 µg/kg. Among them, SHTX-3 was the only one with an antitryptic activity (Table 1), as well as potassium channel blocking activity (Table 2). SHTX-3 toxin has 7035.0 Da and is cross-linked by three disulfide bridges. Its cloned gene encodes a putative sequence of 19 amino acid residues for the signal peptide and 62 for the mature peptide, without a propart between them. Because it was previously proposed that the propart with two basic residues at its end functions as a signal directing toxins into nematocyst [102], it is assumed that SHTX III is not contained in these stinging cnidarian organelles [40]. 

AXPI-I and -II (Table 1) were isolated from the whole body extract of *Anthopleura* aff. *xanthogrammica* [41]. Both are basic peptides, with three disulfide bonds, 58 amino acid residues and belong to the Kunitz-type family. Both inhibited trypsin at a molar ratio of 2:1 (inhibitor: enzyme). AXPI-I was moderately or weakly inhibitory against the serine proteases α-chymotrypsin and elastase, and against the metallo-proteases thermolysin and *Streptomyces griseus* protease. AXPI-II also presented weak inhibition against α-chymotrypsin. Neither of them inhibited the cysteine proteases papain nor bromelain [41]. 

AXAPI, which was another name given to AXPI-I (Table 1), was further isolated from the acrorhagial extract of this same species [24]. Since much higher protease inhibitory activity was displayed by the acrorhagi of *A.* aff. *xanthogrammica* (59 IU/mL) than the remaining tissues (2.9 IU/mL), the authors suggested that AXPI-I is derived from the acrorhagi. Competitive inhibition experiments with ^125^I-α-dendrotoxin and rat synaptosomal membranes suggest that this peptide has no potassium channel toxicity even at 10 µM [24]. In the same study, two Kunitz-type PIs that were purified from the acrorhagial extracts of *Anthopleura fuscoviridis* (AFAPI-I and AFAPI-III, Table 1) and one from *Actinia equina* (AEAPI, Table 1) were also described [24]. Similar to AXAPI, they all inhibited both trypsin and plasmin and did not inhibit potassium channels at the same concentration [24].

The toxin APEKTx1 was isolated from *Anthopleura elegantissima* and is slightly larger than most of the KTTs (Table 1) [42]. With 63 amino acid residues, 7468.5 Da and three disulfide bridges, this toxin inhibits serine proteases and also blocks potassium channels (Table 2), similar to the kalicludines and SHTX-3 [27,40]. APEKTx1 is presumably a competitive trypsin inhibitor, with a dissociation constant (*K*_d_) of 124 nM. However, a 3-fold molecular excess of toxin over trypsin was needed to completely inhibit the protease, which is about 3 times the amount of BPTI or the kalicludines necessary to reach total trypsin inhibition [42]. 

## 3. PIs from *Conus* Venoms

Conotoxins are small disulfide-rich peptide toxins from cone snails usually with 10–30 amino acid residues that have evolved from at least 16 genetically distinct superfamilies, many of which are subject to diverse types of posttranslational modifications, such as *C*-terminal amidation, hydroxyprolines, or glycosylation of serine or threonine [103,104]. They can be divided into several families according to the cysteine bridge pattern and biological activities [104]. In relation to most of *Conus* venom toxins, conkunitzin-S1 (Conk-S1, UniProtKB P0C1X2), from the venom of *Conus striatus*, is unusually long (60 amino acid residues) and has a *C*-terminal amidation as the only posttranslational modification [105]. This potassium channel pore-blocking polypeptide (Table 2) also belongs to the Kunitz-type inhibitor domain, although it is stabilized by only two of the three disulfide bridges normally found in KTTs [105].

The cDNA library constructed with the mRNA from *Conus californicus* venom ducts revealed the presence of four new putative Kunitz-type inhibitors (Cal9.1a to Cal9.1d, UniProtKB D2Y488, D2Y489, D2Y490 and D2Y491, respectively) [106]. The mature peptides of these four compounds have 56 amino acid residues and share high sequence identity. Despite having about the same length of the Conk-S1, these toxins have six cysteine residues. Their activity as protease inhibitors is still uncertain [106]. In addition, another putative Kunitz-type inhibitor (conkunitzin-B1, UniProtKB P0CY85) was obtained from the transcriptome of *Conus bullatus* venom ducts by means of next-generation sequencing techniques [107].

## 4. PIs from Terrestrial Venomous Animals

### 4.1. PIs from Snake Venoms

Similar to those from marine venomous animals, most of protease inhibitors characterized so far from snake venoms present the Kunitz-type motif. These snake venom polypeptides have been demonstrated to specifically inhibit the proteolytic activity of trypsin or chymotrypsin. However, some snake neurotoxins, such as dendrotoxins, calcicludine and B chain of β-bungarotoxin, possess a Kunitz/BPTI-like domain although they exhibit little or no protease inhibitory activity.

In the beginning of 70’s, from the venom of the black mamba (*Dendroaspis polylepis polylepis*), the first trypsin inhibitor homologs (toxins I and K) were reported [108]. Related toxins containing 57–60 amino acid residues and cross-linked by three disulfide bridges were subsequently characterized from different mamba venoms and eventually named dendrotoxins (DTXs), such as the α-dendrotoxin from the green mamba snake (*Dendroaspis angusticeps*) [95,109]. Dendrotoxins are potent blockers of certain subtypes of voltage-gated potassium channels (Table 2) [95,110]. Differently from toxins I and K, dendrotoxin E, from black mamba, was shown to inhibit both trypsin and chymotrypsin (Table 1) [48]. 

Also from *Dendroaspis angusticeps*, calcicludine (CaC, UniProtKB P81658) is a 60-amino acid residue polypeptide that does not display protease inhibitory activity either K^+^ or Na^+^ channel modulator activities. CaC specifically blocks most of the high-threshold Ca^2+^ channels in the nanomolar range, specially the L-type component of the Ca^2+^ current [111,112]. β-Bungarotoxin was previously isolated from *Bungarus multicinctus* venom and comprises two dissimilar polypeptide chains, the A chain (~14 kDa) and B chain (~7 kDa), which are linked by an interchain disulfide bridge [113]. The B chain of β-bungarotoxin is homologous to venom basic protease inhibitors but, similar to most of dendrotoxins, displays no protease inhibitory activity and also blocks voltage-gated K^+^ channels [114].

Despite the dendrotoxin related toxins, several Kunitz-type protease inhibitors have been isolated from snake venoms, particularly from Elapidae and Viperidae snakes. The Russell’s viper venom (RVV) inhibitor, from *Daboia russelii* (=*Vipera russelli*), seems to be the first of them [101]. It is a basic polypeptide with deduced 60 amino acid residues and inhibitory activity against plasma and pancreatic kallikreins, plasmin and trypsin, being more potent against trypsin. A Kunitz-type inhibitor homolog (RVV inhibitor II, UniProtKB P00990) was further sequenced from the venom of the Eastern Russel’s viper (=*Daboia siamensis*) [115]. Recently, similar polypeptides were identified in the venom of *Daboia siamensis* [49,50]. From the Burmese specie, BBPTI-1 (Table 1) was shown to strongly inhibit chymotrypsin activity, with no detectable inhibitory activity against trypsin [49]. From the Chinese one, two trypsin inhibitors (CBPTI-1 and CBPTI-2, Table 1) and one chymotrypsin inhibitor (CBPTI-3, Table 1) were purified and cloned [50].

Five PIs were isolated from the venoms of the African Elapids *Hemachatus haemachatus* and *Naja nivea* [51]. The isolated trypsin inhibitors have 52 to 57 amino acid residues, and were all devoid of tryptophan. Except for one peptide from *Naja nivea* venom, designated NNV inhibitor la, which had two disulfide bonds, all the others contained three. Two high homologous peptides (HHV inhibitor II and NNV inhibitor II, Table 1) were completely sequenced. Similar to HHV inhibitor I, HHV inhibitor II was active against trypsin, α-chymotrypsin, plasma kallikreins and plasmin from bovine sources. NNV inhibitor II was only tested against trypsin.

From the venom of the long-nosed viper *Vipera ammodytes*, three basic PIs were isolated [52]. Two of them strongly inhibit trypsin, whereas the third one primarily inhibits chymotrypsin (Table 1). Other enzymes were also inhibited, although with lower potency. The precursors of the chymotrypsin inhibitor and trypsin inhibitor I were further obtained [116]. An homologous putative trypsin inhibitor was also sequenced from the venom of the leaf-nosed viper, *Eristicophis macmahoni* (UniProtKB P24541) [117].

Until now, two PIs have been isolated from *Bungarus fasciatus* venom [54,55]. The first of them was denoted protease inhibitor IX (or BF9, Table 1) and consists of a polypeptide chain of 65 amino acid residues that, when cleaved, generates the protease inhibitor VIIIb [54]. BF9 specifically inhibits α-chymotrypsin in a 1:1 molar ratio and is structurally similar to the Kunitz-type toxins [54,118]. The second is a Kunitz-type serine protease inhibitor named bungaruskunin (Table 1), which was characterized from venom and also cloned from venom glands [55]. The predicted precursor contains 83 amino acid residues, including a 24-residues long signal peptide. The mature bungaruskunin inhibits trypsin, chymotrypsin, and elastase, but not thrombin. 

From the venom of the cobra *Naja naja*, a strong trypsin inhibitor (Table 1) [56] and a putative 57-amino acid residue chymotrypsin inhibitor (UniProtKB P19859) were characterized [119]. With almost 90% identity to the latter, a chymotrypsin inhibitor was also characterized from the venom of *Naja atra* (NACI, Table 1) [57]. No detectable inhibitory effects were observed on trypsin or plasmin. Its precursor was further characterized and it was shown that the expressed protein without the first three residues at its *N*-terminal led to a moderate increase in the inhibitory constant against chymotrypsin [58].

A weak chymotrypsin inhibitor with no inhibitory action against trypsin was characterized from the venom of the king cobra *Ophiophagus hannah* (Oh11-1, Table 1) [59]. After that, a peptide named OH-TCI was shown to inhibit both chymotrypsin and trypsin with similar potencies [60]. The inhibition constants of the native peptide for chymotrypsin and trypsin were both around 170 nM, while the recombinant peptide presented no more than 5-fold difference between both values (Table 1). No detectable effects were observed against thrombin and subtilisin [60].

The structural organization of the genes encoding three protease inhibitor-like proteins (PILP-1 to PILP-3) from *Bungarus multicinctus* was reported [61]. Recombinant PILP-1 inhibited trypsin (Table 1), whereas PILP-2 (UniProtKB B4ESA3) and PILP-3 (UniProtKB B4ESA4) did not inhibit chymotrypsin or trypsin. PILP genes share identical organization with B chain genes, with three exons interrupted by two introns in similar positions. Comparative analysis of PILP and B chain genes, together with the high degree of sequence similarity of the polypeptides, suggested that these genes have originated from a common ancestor and that they should be evolved by gene duplication followed by divergence [61]. 

Two distinct types of KTTs have already been characterized from the venom of Australian elapid snakes. Taicatoxin serine protease inhibitor (TSPI, Table 1) was purified from the venom of *Oxyuranus scutellatus scutellatus* as part of a multimeric complex that blocks high threshold calcium channels and small conductance Ca^2+^-activated K^+^ channels [62,120]. In its isolated form, TSPI exhibits antifibrinolytic activity and is a broad spectrum inhibitor, being active against plasma and tissue kallikrein, trypsin, elastase, factor Xa, and α-factor XIIa [62,63]. Subsequently, a second family of Kunitz-type inhibitors was purified, cloned and characterized from the venom of the common brown snake *Pseudonaja textilis* [7,121]. Two of them, textilinin-1 and -2 (Table 1), were shown to be potent plasmin inhibitors [7,63]. Textilinin-1 is also a potent trypsin inhibitor, with little effect on both plasma and tissue kallikrein, and no effect on many other proteases tested [63,122]. Because textilin-1 has been shown to inhibit plasmin and reduce bleeding in a small animal model [7], further studies have suggested its use as a therapeutic alternative for aprotinin [122,123], which is widely used in surgery as an anti-bleeding agent but is associated with numerous side effects [124].

From the New Guinean *Pseudechis australis* (=*Pseudechis rossignolii*), three cDNAs encoding Kunitz-type protease inhibitors were isolated and named *Pr*-mulgins 1, 2 and 3 [65]. The putative polypeptides are 92.4%–99.3% identical with their orthologs in the Australian *Pseudechis australis* [125]. Recombinant *Pr*-mulgin 1 significantly affected matrix metalloprotease (MMP) 2; *Pr*-mulgin 2 and 3 strongly inhibited trypsin and plasma plasmin; and *Pr*-mulgin 2 also inhibited α-chymotrypsin (Table 1). No inhibitory effects were observed against other proteases such as serine proteases (elastase, kallikrein and pepsin), cysteine protease (cathepsin G), and MMPs (1,3,7,8,9,10,12,13 and 14). These peptides did not block *Drosophila* K^+^ channels (*Shaker*) and rat K^+^ channels (K_v_1.1).

Recently, a trypsin inhibitor named PIVL (Table 1) was characterized from the venom of the Tunisian snake *Macrovipera lebetina transmediterranea* [64]. Besides trypsin inhibitory activity, PIVL also exhibits anti-tumor effect and displays integrin inhibitory activity without being cytotoxic. No inhibitory effect was detected on chymotrypsin. 

Transcriptomic approaches are also showing numerous serine PIs in snake venoms. Transcriptome of the venom glands of the Australian elapid snake *Drysdalia coronoides* revealed that serine protease inhibitors are one of the major components from this venom [126]. The presence of putative Kunitz-type inhibitors was also shown in the venom gland transcriptomes of the elapid snakes *Bungarus multicinctus* and *Naja atra* [127], of the viper snake *Vipera nikolskii* [128], of the Brazilian coral snake *Micrurus altirostris* [129], and from several Australian elapid snakes [125].

Other classes of PIs in snake venoms comprise the cystatins and the bradykinin-potentiating peptides (BPPs). The cystatins are a family of cysteine protease inhibitors with homology to chicken cystatin/ovocystatin and human cystatin C [130]. To date, few snake venom cystatins have been fully characterized, and some of them were shown to inhibit papain-like cysteine proteases [131,132,133,134]. BPPs consist of about 5–14 amino acid residues that specifically block the endothelial metalloprotease angiotensin I-converting enzyme (ACE). The first report of a “bradykinin-potentiating factor (BPF)” in the venom of *Bothrops jararaca* was in 1965 [135]. These factors were subsequently purified and characterized as peptides responsible for potentiating the activity of bradykinin and inhibition of ACE [136,137]. Among a series of analogs of BPPs studied, one of them was modified to produce captopril, an orally available peptidomimetic that, together with further modified molecules, become widely used for the treatment of hypertension [138]. Although not presented in detail in this review, other BPPs have been identified in snake venoms or venoms glands [139,140,141,142,143].

### 4.2. PIs from Scorpion Venoms

The first report on a protease inhibitor from scorpion venom was made in 1981 by Chhatwal and Habermann [66]. The peptide was purified from the venom of the Indian red scorpion *Mesobuthus tamulus* and consisted of 77 amino acid residues and 8500 Da (Table 1). It inhibited trypsin and also kallikrein, but had weak or no activity against chymotrypsin, plasmin and thrombin [66]. Its sequence, however, was not revealed. 

Posteriorly, Schwartz and collaborators [67] reported for the first time, by means of transcriptomic analysis, the presence of a Kunitz type precursor in scorpions. The mature peptide, named Hg1, from the Mexican scorpion *Hadrurus gertschi*, was not found in the venom and its activity remained unknown, until it was recently expressed by *Escherichia coli* BL21(DE3) cells, purified and tested [68]. Together with Hg1, other six recombinant scorpion peptides (LmKTT-1a, LmKTT-1b, LmKTT-1c, BmKTT-1, BmKTT-2 and BmKTT-3) were also characterized as trypsin inhibitors (Table 1), with no activity against chymotrypsin or elastase even at high doses [68]. Among them, recombinant Hg1 presented the lowest dissociation constant against trypsin, while rBmKTT-3, from *Mesobuthus martensii*, had the highest [68]. LmKTT-1b, also named SdPI, from *Lychas mucronatus*, was the first functionally characterized Kunitz-type peptide from scorpion [16]. All these seven recombinant peptides seem to have similar secondary structure to BPTI, as analyzed by circular dichroism spectroscopy [68]. Moreover, some of them are also blockers of potassium channels (Table 2) [68].

From the cDNA libraries constructed from the venom glands of three scorpion species, four putative serine protease inhibitors belonging to the *Ascaris*-type peptides were recently identified: SjAPI (*Scorpiops* *jendeki* *Ascaris*-type protease inhibitor), SjAPI-2 (*Scorpiops* *jendeki* *Ascaris*-type protease inhibitor 2), CtAPI (*Chaerilus tricostatus Ascaris*-type protease inhibitor), and BmAPI (*Buthus martensii Ascaris*-type protease inhibitor) [69]. Members of the *Ascaris*-type peptides’ family are characterized by the presence of five disulfide bonds [144]. The recombinant SjAPI (Table 1) inhibited both α-chymotrypsin and elastase, with no detectable effect on trypsin. SjAPI is produced as a precursor polypeptide of 94 amino acid residues, which includes a signal peptide of 24 residues, a propeptide of 6 residues, and a 64-residue mature peptide [69]. 

Besides these serine protease inhibitors, a plasmin inhibitor named discreplasminin was isolated from *Tityus discrepans* scorpion venom. It has an antifibrinolytic mechanism similar to aprotinin and probably interacts with the active sites of plasmin and tPA, a tissue-type plasminogen activator [145]. Moreover, a putative serpin peptidase inhibitor-like protein (UniProtKB C5J8C1) was identified in the cDNA venom gland library of *Opisthacanthus cayaporum*. However, it is composed by 176 encoded amino acid residues and lacks the signal peptide region [146].

### 4.3. PIs from Spider Venoms

Yuan and collaborators reported the first superfamily of Kunitz-type toxins from spiders [14]. It is composed by HWTX-XI, the prototype member of this superfamily, and 45 additional putative KTTs, which were found by means of two venom gland cDNA libraries constructions of the two Chinese bird spiders *Ornithoctonus huwena* and *O. haiana* (34 and 11 putative KTTs, respectively). HWTX-XI (Table 1) has 6166.23 Da and six cysteine residues. It was isolated from both venom and venom gland extract of *O. huwena*. HWTX-XI inhibits trypsin stoichiometrically in a 1:1 ratio and strongly binds to trypsin, while presenting lower affinity to α-chymotrypsin. It had about 30-fold higher inhibition potential to trypsin compared with BPTI, with a *K*_d_ value of 2.3 × 10^−10^ M, while BPTI had a *K*_d_ of 6.57 × 10^−9^ M under the same experimental conditions [14]. Similar to many KTTs, HWTX-XI also inhibits potassium channel currents (Table 2).

After that, a putative serine protease inhibitor was revealed from the cDNA library of *Loxosceles intermedia* venom gland [147]. This single EST (cluster LIS209) presented high similarity with serine protease inhibitors from mammals, such as *Mus musculus* (UniProtKB O35684) and *Pan troglodytes*, and also from *Ambliomma americanum* tick. 

Recently, the first spider Kunitz-type inhibitor with plasmin and elastase inhibitory activity (AvKTI, Table 1) was identified in *Araneus ventricosus* [70]. However, its coding clone was selected from the expressed sequence tags (ESTs) that were generated from a cDNA library constructed using *A. ventricosus* whole bodies. Because AvKTI is expressed only in the epidermis, it is suggested that it is a Kunitz-type inhibitor derived from the spider body, but not from venom [70]. Recombinant AvKTI inhibited both trypsin and chymotrypsin, with no detectable inhibitory effects on factor Xa, thrombin or tissue plasminogen activator (tPA). However, it inhibited plasmin and neutrophil elastase [70]. Thus, among the proteases tested, the most prominent inhibitory effect of AvKTI was observed on plasmin, resembling other PIs with antifibrinolytic activity, such as scorpion discreplasminin [145] and snake textilinins [7,122]. In addition, another Kunitz-type inhibitor (AvCI, Table 1) was obtained from *A. ventricosus* in a similar manner to AvKTI [71]. AvCI inhibits chymotrypsin, the microbial serine proteases subtilisin A and proteinase K, and also the human neutrophil elastase and porcine pancreatic elastase. It was ineffective against trypsin, factor Xa, thrombin, tPA and plasmin [71].

### 4.4. PIs from the Skin Secretion of Anurans

The skin granular glands of Anurans (frogs and toads) produce a remarkably diverse range of bioactive polypeptides [17]. Among them, almost all the serine protease inhibitors from amphibians contain 3–5 conserved disulfide bridges, with few exceptions [85,86,87], and their biological role is still not clear [87]. Unless otherwise stated, all amphibian PIs described in this section were obtained from their skin secretions.

From the toad *Bombina bombina*, a 60-residue polypeptide cross-linked by five disulfide bridges was isolated and cloned [72]. Named BSTI, it inhibits both porcine trypsin and human thrombin (Table 1), with no activity against chymotrypsin. Its precursor contains 84 amino acid residues, including a 24-residue signal peptide [72]. Structurally homologous to BSTI, a trypsin inhibitor named BMTI (Table 1) was characterized from the toad *Bombina maxima* [73]. Its precursor also contains 84 amino acid residues, with a putative signal peptide of 24 residues. Unlike BSTI, BMTI had no inhibitory effect on thrombin. Chymotrypsin, elastase and bacterial subtilisin were also not inhibited at up to 10 µM concentration. Also with five disulfide bridges, a weak trypsin inhibitor was isolated from the frog *Rana areolata* (Table 1) [74].

In addition, two trypsin inhibitor analogs (BOTI and BVTI) were isolated from the toads *Bombina orientalis* and *Bombina variegata* (Table 1) [75]. By means of a new non-invasive technique, two cDNA libraries were constructed from the dermal venom of these two species and both precursors were obtained, which contain 84 amino acid residues, including a 24-residue signal peptide. The trypsin inhibitory activity of BOTI and BVTI was not measured [75]. 

From the toad *Bombina microdeladigitora*, two serine protease inhibitors structurally homologous to BSTI were identified (BMSI 1 and BMSI 2, Table 1) [76]. BMSI 1 inhibits both trypsin and thrombin. No inhibition of the hydrolysis of substrates by elastase, chymotrypsin and subtilisin were observed with an inhibitor concentration up to 10 µM.

From *Hyla simplex*, two serine protease inhibitors were isolated (Table 1)—an alpha-1-antitrypsin-like serpin (hylaserpin-S1) and a wasp venom-like toxin (hylaserpin-S2) [77]. Hylaserpin-S2 precursor is composed by 83 amino acid residues, including a predicted signal peptide of 27 residues. It is structurally related to those amphibian serine protease inhibitors containing five disulfide bridges from *Bombina* and *Rana* genera [72,73,74,75]. Hylaserpin-S1 is a much longer peptide, with 44 kDa, and is encoded by a 415-amino acid residues precursor, with a signal peptide of 23 amino acid residues. Hylaserpin-S1 inhibited both trypsin and chymotrypsin, whereas hylaserpin-S2 only inhibited trypsin, similarly to BSTI [72,77]. Moreover, hylaserpin-S2 had bacteriostatic effect against Gram-positive bacteria *Bacillus subtilis*, and hylaserpin-S1 displayed direct microorganism-killing abilities against *B. subtilis*, *E. coli*, and *Candida albicans* [77].

Another long protease inhibitor, consisting of a basic single chain glycoprotein with about 22 kDa, was isolated from the toad *Bufo andrewsi* (BATI, Table 1). BATI inhibits trypsin, but displays no inhibitory activity against chymotrypsin, thrombin and elastase [78]. Also from *Bufo andrewsi*, an irreversible serine protease inhibitor (baserpin, Table 1) also consisting of a single chain glycoprotein with ~60 kDa was purified. Baserpin, which possibly belongs to the serpin superfamily, inhibits the catalytic activity of trypsin, chymotrypsin and elastase, with no detectable inhibitory effects on thrombin [79]. Similar to BATI, the peptide KPHTI (Table 1), from the frog *Kaloula pulchra hainana*, is a single chain glycoprotein with about 23 kDa, and also inhibits trypsin, but not chymotrypsin, thrombin, elastase or subtilisin [80]. Only few *N*-terminal residues from BATI, baserpin and KPHTI were sequenced [78,79,80].

An albumin-like protein with approximately 60 kDa (albumin-1, UniProtKB Q3T479) was isolated and cloned from *Bombina maxima* skin [148]. Purified from skin homogenate, this protein inhibits trypsin (*K*_d_ of 1.92 nM), with no inhibition against thrombin, chymotrypsin, elastase or subtilisin. However, this protein, which shares similar biochemical and immunochemical properties as those of the *B. maxima* serum albumin, was not detected in the skin secretions [148]. 

Two peptides analogous to the Kazal family of serine protease inhibitors were obtained from the skin extract of *Phyllomedusa sauvagii* (PSKP-1 and PSKP-2, Table 1) [81]. Recombinant PSKP-1 inhibited a serum prolyl endopeptidase from blood serum, but was not active against trypsin, chymotrypsin, V8 protease or proteinase K. In addition, PSKP-1 displays bactericidal activity and induces agglutination of red cells and bacteria [81]. Similar to these peptides, two putative protease inhibitors (PI01 and PI02) were obtained from the cDNA library of *Phyllomedusa nordestina* (Table 1). The third of them (PI03) is shorter and structurally different [82]. A different Kazal-type protein with potent trypsin inhibitory activity (ACKTI, Table 1) was characterized from the frog *Agalychnis callidryas* [83]. Its precursor contains 78 amino acid residues, including a signal peptide with 26 residues.

A Kunitz-type protease inhibitor (Table 1) was isolated from the skin secretion of the tomato frog *Dyscophus guineti* [17]. It contains 57 amino acid residues, including six cysteine residues, and inhibits trypsin. In addition, a chymotrypsin inhibitor of Kunitz-type (KSCI, Table 1) was characterized from *Kassina senegalensis* [84]. Its precursor sequence contains 84 amino acid residues, including a 22-residue putative signal peptide.

From the frog *Hyla annectans*, a different Kunitz-type inhibitor peptide (anntoxin, Table 1) with additional (although weak) sodium channel activity was characterized [85]. It inhibits tetrodotoxin-sensitive (TTX-S) sodium channel currents elicited from adult rat dorsal root ganglion neurons with an IC_50_ value of 3.4 µM, and is also lethal to different potential predators, like insects, snake, birds, and mice. Calcium and potassium currents were few or not inhibited even at concentrations up to 10 µM. Anntoxin inhibits trypsin and is homologous to KTTs, but contains only two of the three highly conserved disulfide bridges [85]. 

The first small serine protease inhibitor known to contain only a single disulfide bond characterized from animals was isolated and cloned from the skin secretion of the frog *Odorrana grahami* [86]. Five different precursors, although highly conserved, encode OGTI. All of them contain 70 amino acid residues including a signal peptide of 22 residues, an acidic spacer peptide with 31 residues and equal mature peptides with 17 residues. OGTI toxin inhibited trypsin (Table 1), but had no inhibitory effects on thrombin, chymotrypsin, elastase, subtilisin, plasmin or furin at concentrations up to 10 µM [86]. Similar precursors encoding mature peptides equal to OGTI seem to have been found in the skin secretions of *Odorrana andersonii* (UniProtKB E3SZL1), *Odorrana margaretae* (UniProtKB E1AWD0) and *Rana schmackeri* (UniProtKB D5LXG2).

Another small peptide with only one disulfide bond (HV-BBI, Table 1) was characterized from the frog *Huia versabilis* (=*Odorrana versabilis*) [87]. This Bowman-Birk type protease inhibitor is encoded by a precursor of 62 amino acid residues, including a 22-residue signal peptide, a 22-residue propeptide, and a mature peptide consisting of 18 amino acid residues and a *C*-terminal amidation. The synthetic peptide was found to behave as a competitive reversible inhibitor of trypsin. No chymotrypsin inhibitory activity was observed [87].

### 4.5. PIs from Hymenopterans’ Venoms

There are few reports on serine protease inhibitors from the venom of Hymenopterans, such as wasps and bees. Moreover, some of them are putative PIs, obtained by means of transcriptomic tools, and have not been tested yet. 

From the venom of the solitary spider wasp *Anoplius samariensis*, a peptide named As-fr-19 (UniProtKB Q589G4) was purified and cloned [149]. Its precursor encodes a peptide with 75 amino acid residues, containing a signal peptide of 17 residues and a mature toxin of 58 residues. As-fr-19 contains six cysteine residues and presents sequence similarity to some sea anemone and snake toxins, such as the kalicludines from *Anemonia sulcata* [27] and snake dendrotoxin I and K and calciludine [108,111]. Thus, As-fr-19 may exert inhibitory effects on serine proteases and on potassium and/or calcium channels [149].

Using random sequencing analysis, novel putative toxins from the venom of the parasitoid wasp *Pimpla hypochondriaca* have been sequenced [150]. Among the *c*ysteine-rich *v*enom *p*roteins, four of them (cvp1, cvp2, cvp4 and cvp6) presented sequence similarity with protease inhibitors. Both cvp1 and cvp6 contain 10 cysteine residues and are similar to the chymotrypsin inhibitor AMCI 1 from the larval hemolymph of *Apis mellifera* (UniProtKB P56682). Mature cvp2 shares high sequence similarity to Kunitz-type serine protease inhibitors, and cvp4 consists of a three times repeated six cysteine motif and is similar to pacifastin, a protease inhibitor from locust (UniProtKB Q8WQ22). 

A serine protease inhibitor polypeptide named bicolin (Table 1) was characterized from the venom of the wasp *Vespa bicolor* Fabricius [88]. Its precursor is composed of 77 amino acid residues comprising a 23-residue predicted signal peptide and the 54-residue mature toxin cross-linked by three disulfide bonds. Bicolin is homologous to As-fr-19 and cvp2 and showed inhibitory activity against trypsin and thrombin, but had no effect on elastase and chymotrypsin. Bicolin was found to own anticoagulation function, possibly due to its thrombin-inhibitory activity [88].

From *Bombus ignitus* bumblebee venom, a Kunitz-type serine protease inhibitor (Bi-KTI, Table 1) with plasmin inhibitory activity was characterized [6]. Its precursor consists of 82 amino acid residues, with a predicted 24-residue signal peptide and a 58-residue mature peptide, including six cysteine residues. It strongly inhibits plasmin, although its inhibitory activity was approximately two-fold weaker than that of aprotinin. Bi-KTI did not inhibit other enzymes from the hemostatic system, such as factor Xa, thrombin, or tPA. These results are similar to those of snake textilinins [7,122]. Bi-KTI, which acts as an antifibrinolytic agent, and Bi-VSP, a *Bombus ignitus* venom serine protease previously characterized that acts as a fibrin(ogen)olytic agent [151], may act in a cooperative fashion to promote the spread of bee venom under anti-bleeding conditions [6].

Similar results were obtained with the Kunitz-type inhibitor Bt-KTI (Table 1), from *Bombus terrestris* bumblebee venom [89]. Bt-KTI shares high sequence similarity to Bi-KTI and also consists of a 58-amino acid residue mature peptide, including six conserved cysteine residues. Moreover, it displays strong inhibitory activity against plasmin, exhibiting an antifibrinolytic activity, and does not inhibit factor Xa, thrombin or tPA [89].

From a cDNA library constructed using Asiatic honeybee (*Apis cerana*) whole bodies, a putative chymotrypsin inhibitor (AcCI) with ten conserved disulfide bridges was identified [152]. Based on the RT-PCR done with samples of epidermis, fat body, midgut, and venom gland, the AcCI gene was found to be constitutively expressed in all of these tissues. The recombinant AcCI (rAcCI), obtained by a baculovirus/insect cell expression system, inhibited the activity of chymotrypsin (IC_50_: 24.71 nM), with an inhibitory constant (*K*_i_) of 11.27 nM, and of human neutrophil (IC_50_: 38.50 nM) and porcine pancreatic (IC_50_: 70.21 nM) elastases, with *K*_i_ values of 61.05 nM and 101.89 nM, respectively. rAcCI had no inhibitory activity against trypsin, factor Xa, thrombin, tPA, or plasmin. It is worthy to mention that although AcCI precursor codes to a 65-amino acid residue mature peptide, with a predicted molecular mass of 7.2 kDa, rAcCI was identified as a 16-kDa protein, which was interpreted by authors as due to the presence of carbohydrate moieties [152].

## 5. PIs and Potassium Channel Activity

It is suggested that the evolution process of Kunitz-type toxins has probably three stages: old functional molecules, bi-functional toxins and new function toxins [14]. Thus, some Kunitz-type protease inhibitors could acquire the neurotoxin function, whereas others would even lose their protease inhibitory role and act on voltage-gated ion channels [14,116]. Potent and specific neurotoxic K^+^ channel blockers with Kunitz-type motif are particularly developed in snakes, while sea anemone, scorpion and spider toxins have developed only the dual-functional KTTs, frequently with weak K^+^ channel blocking activity. One possible explanation is that the selective pressure would have act in order to keep the dual-functional KTTs with weak K^+^ channel blocking activity in these latter animals, which already have other potent neurotoxins in their venoms/body extracts. By contrast, only K^+^ channel blockers presenting the Kunitz-type motif were characterized from snake venoms so far [14]. 

Typical examples of this are the snake dendrotoxins. Although being similar to Kunitz-type protease inhibitors in amino acid sequences and three-dimensional conformation [153], dendrotoxins display few or no serine protease inhibitory activity. By contrast, they are strong blockers of potassium channels (Table 2) [95,110]. Both dendrotoxin (or α-dendrotoxin, UniProtKB P00980) from green mamba *Dendroaspis angusticeps* and toxin I (UniProtKB P00979), from the black mamba *Dendroaspis polylepis*, block cloned K_v_1.1, K_v_1.2 and K_v_1.6 channels in the low nanomolar range (*K*_d_ < 20 nM) [95,154]. Toxin K (UniProtKB P00981), also from the black mamba *D. polylepis*, preferentially blocks K_v_1.1 channels and is active at picomolar concentrations [96,155]. Other subtypes of potassium channels are also blocked by some dendrotoxins, although with lower affinity [95]. The protein E homologues from green mamba venom are also able to block K_v_1.1 channels expressed in oocytes, although higher concentrations are needed (Table 2) [98]. Named “DaE1” and “DaE2”, these polypeptides share 98% and 95% identity, respectively, to trypsin inhibitor E from black mamba venom [156].

By means of chemical modifications of native dendrotoxins and genetic engineering to produce mutated toxins, the amino acid residues that are essential for their interaction with potassium channels have been identified [95,110]. Lys5 seems to be the major determinant of the binding affinity of α-DTX and DTX-I for K^+^ channels (Figure 1) [157,158]. Along with it, the neighbor hydrophobic residue Leu9 is also important for binding to K^+^ channels [159]. Lys19, which is around the “anti-protease” site, caused only a little loss of activity when acetylated in DTX-I [158] or replaced by Ala in α-DTX [159]. 

Besides Lys5, or the equivalent Lys3 in DTX-K, the amino acids at the β-turn region (residues 24–28 in toxin K, in particular Trp25 and Lys26) are responsible for the potassium channel activity [160,161]. In DTX-I, acetylation of Lys29 inactivated the toxin, possibly by producing large structural perturbations [158]. Moreover, although acetylation of Lys28 alone had little effect, the toxin became almost inactive when both Lys28 and Tyr24 were modified [158]. Differently, in α-DTX, modification of the Lys triplet 28–30 to Ala-Ala-Gly exhibited only small decreases in biological activity [162]. Seven amino acid residues (Lys3, Tyr4, Lys6, Leu7, Pro8, Arg10 and Lys26) from δ-dendrotoxin were shown to be important for the toxin’s interaction with a *Shaker* channel variant [163]. 

The heterodimeric snake neurotoxin β-bungarotoxin inhibits the release of acetylcholine from motor nerve endings [164]. It consists of a PLA_2_ subunit and a K^+^ channel binding subunit, which is a member of the Kunitz-type protease-inhibitor superfamily, both linked by a disulfide bridge [165]. β-Bungarotoxin acts on a presynaptic potassium channel and then, with the phospholipase A_2_ unit activity, blocks neurotransmission, altering the acetylcholine release [166]. β-Bungarotoxin (2 µM/L) partially blocked fast K^+^ outward current (I_K.F_) through the fast K^+^ channels and also Ca^2+^-dependent K^+^ current (I_K(Ca)_) through Ca^2+^-activated K^+^ channel in motor nerve terminals of snake [167]. The mutated B(C55S)-bungarotoxin chain, where Cys55 was replaced by Ser55, at 200 nM, blocked the outward K^+^ current through synaptosomal membranes. However, the B chain is not the only essential subunit for the binding of β-bungarotoxin to its target given that it has been shown that Ca^2+^ is required for the binding of β-bungarotoxin with its receptors [168] and that the A chain is the Ca^2+^-binding subunit of the toxin [169].

Some dendrotoxin homologues have been characterized in other venomous animals, such as sea anemones, cone snails, scorpions and spiders (Figure 1 and Table 2), raising the possibility that the protease inhibitor structural framework has been used to create potassium channel blocking activity, being the new active site different from and independent of the old one [14].

The kalicludines AsKC1, AsKC2 and AsKC3, from the sea anemone *Anemonia sulcata*, were shown to inhibit the binding of ^125^I-α-dendrotoxin to rat brain membranes in a competitive way, with IC_50_ values of 375 nM for AsKC1 and 500 nM for AsKC3. AsKC2 had an inhibition constant *K*_i_ of 20 nM. All these toxins were able to inhibit K_v_1.2 channels expressed in *Xenopus* oocytes (Table 2) [27]. Similarly, SHTX-3, from the sea anemone *Stichodactyla haddoni*, inhibited the binding of ^125^I-α-dendrotoxin to rat synaptosomal membranes [40]. Its IC_50_ value (Table 2) indicated that this toxin is about 110 times less potent than α-dendrotoxin, which under the same conditions presented an estimated IC_50_ value of 5.7 nM.

The sea anemone peptide APEKTx1, from *A. elegantissima*, was subject to a wide screening on 23 ion channels expressed in *Xenopus laevis* oocytes: 13 cloned voltage-gated K^+^ channels (K_v_1.1–K_v_1.6, K_v_1.1 triple mutant, K_v_2.1, K_v_3.1, K_v_4.2, K_v_4.3, hERG, the insect channel *Shaker* IR); 2 cloned hyperpolarization-activated cyclic nucleotide-sensitive Ca^2+^ non-selective channels (HCN1 and HCN2) and 8 cloned voltage-gated Na^+^ channels (Na_v_1.2–Na_v_1.8 and the insect channel DmNa_v_1) [42]. With exception of K_v_1.1 channel, no significant effects could be observed on the other ion channel isoforms at concentrations up to 1 µM. The IC_50_ value for K_v_1.1 channel (Table 2) indicated that APETx1 inhibits K_v_1.1 channels with the same potency as DTX-I and α-DTX. The inhibition of K_v_1.1 channels by this toxin is reversible and not voltage-dependent. APETx1 did not alter channel gating and it presumably acts by blocking the pore in the open state of the K_v_ channel. It is suggested that APEKTx1 acts on the channel’s extracellular site [42].

It was investigated whether the sensitivity of APEKTx1 for K_v_1.1 channels could be affected by mutating 3 amino acid residues (A352P, E353S and Y379H) in the dendrotoxin binding site, which is located in the H5 loop between the transmembrane domains S5 and S6 of K_v_1.1 α-subunit. By doing these mutations, the pore region of this triple mutant closely resembles the one of K_v_1.3 channels. Even at 100 µM concentration, APEKTx1 only induced 53% current inhibition at the mutated channel, yielding an IC_50_ value of 10.8 ± 0.6 µM, highlighting the crucial interaction of these channel residues with the toxin. The same experiment was performed with DTX-K and it was obtained an IC_50_ value of 0.51 ± 0.064 µM for wild-type K_v_1.1 channels and 5.28 ± 0.23 µM for the triple mutant channel, which represents a 10-fold decrease in sensitivity [42]. 

Despite the high sequence similarity between InhVJ toxin, from the sea anemone *A. sulcata*, and these other sea anemone polypeptides (up to 50%), InhVJ did not modified potassium channel currents (K_v_1.1–K_v_1.6, *Shaker*, K_v_2.1, K_v_3.1, K_v_4.2, K_v_4.3 and cardiac hERG channel) expressed in *Xenopus* oocytes in a concentration range of up to 50 µM [47]. This inactivity was attributed mainly to the absence of a functional dyad consisting of basic and hydrophobic amino acid residues in the structure of InhVJ. In addition, it was suggested that the small positive charge (+0.02) of InhVJ was apparently not enough to inhibit K_v_ channels, since heavily charged polypeptides would interact better with the negative electrostatic potential of the extracellular part of potassium channels [47]. 

Recombinant conkunitzin-S1, from the cone snail *Conus striatus*, was shown to inhibit the *Shaker* potassium channel expressed in *Xenopus* oocytes (Table 2) [94,105]. Conkunitzin-S1presented high affinity for mutated K427D *Shaker*-Δ6-46 channels, with an IC_50_ value that was in good agreement to that of Conk-S1 obtained from natural source [94]. 

All seven scorpion protease inhibitors described by Chen and collaborators [68] were tested on K_v_1.3 channels (Table 2). The toxins rLmKTT-1a, rLmKTT-1b and rLmKTT-1c inhibited ~50% of K_v_1.3 channel currents at 1 µM concentration, whereas rHg1, rBmKTT-1 and rBmKTT-2 promoted ~60%–80% current inhibition at the same concentration. rHg1 was also active at 100 nM concentration, and rBmKTT-3 had only a weak effect on K_v_1.3 channel currents. rHg1 was also shown to inhibit <50% of the K_v_1.1 and K_v_1.2 channel currents, with little effect on SKCa3 and BKCa channel currents, all at 1 µM concentration. These results indicate that Hg1 is specific for K_v_1.3 channels [68]. 

Different Hg1 mutants (generated by the alanine-scanning strategy) were tested on K_v_1.3 channels [68], showing no apparent effects of His2, His3, Asn4, Arg5, Leu9 and Lys13 residues on the pharmacological activity of the toxin. Since basic amino acid residues from animal toxins are usually essential for the potassium channel blocking activity [172,173], mutations were performed on the second cluster of basic residues of Hg1, located at the *C*-terminus of the toxin. Replacement of Lys56, Arg57, Phe61 and Lys63 residues (Figure 1) by alanine significantly reduced the K_v_1.3 blocking activity of mutants by about 94-, 49-, 58- and 74-fold, respectively. Since no molecular conformational changes were observed by circular dichroism analysis between mutants and native peptide, these data indicate that Hg1 mainly uses its *C*-terminal residues, and not those from its *N*-terminal, to inhibit the K_v_1.3 channel. This is different from the known mechanism of the KTTs such as HWTX-XI and δ-dendrotoxin, which use their *N*-terminal residues to block K_v_1.1 channels [14,174]. A structural model of the Hg1-K_v_1.3 complex was computationally obtained, supporting the importance of these four *C*-terminal residues of Hg1 as the channel-interacting surface. Lys56 is the pore-blocking residue; Phe61 contacts residues of the channel A and D chains; and Arg57 and Lys63 mainly contacts residues of the channel D chain [68]. 

The spider venom toxin HWTX-XI inhibited potassium channels expressed in rat dorsal root ganglion neurons, reducing the amplitude of potassium currents by 41.7% ± 1.8% at 1 µM concentration [14]. Inhibition was voltage- and concentration-dependent, with an IC_50_ value of 11.6 nM. Experiments with K^+^ channel subtypes expressed in *X. laevis* oocytes showed that HWTX-XI was more active on K_v_1.1 channels (78% ± 7% inhibition) than on K_v_1.2 and K_v_1.3 (10% ± 2% and 28% ± 3% inhibition, respectively), although a high dose (5 µM) was needed (Table 2) [14]. 

Studies with 18 mutants of HWTX-XI [14], which were constructed through site-directed mutagenesis using HWTX-XI gene as template, showed that the residue Leu6 seems to be essential for the K^+^ channel blocking activity (Figure 1). Its mutation to Ala or Tyr produced about 200-fold reduction in the inhibitory potency. The residues Arg5 and Arg25 seem to have a secondary role in the blocking function, once the mutations R5I and R25A produced about 14 and 4-fold reduction in the potassium channel activity, respectively. 

Comparisons between the primary (Figure 1) and tertiary structures of HWTX-XI and DTX-K provided some clues to explain why HWTX-XI is weaker than DTX K for the K_v_ channel blocking activity [14]. It was shown that the key residue for channel binding Lys3 in DTX-K is replaced by Asp2 in HWTX-XI, which is negatively charged and has a shorter side chain. Although this amino acid residue may contribute to the smaller blocking activity of HWTX-XI, the mutation D2K in this molecule increased the activity by only about 4-fold. It is possible that the side chain of Lys at the mutant was not at the molecular surface. Another difference between the two compounds is that HWTX-XI does not possess the equivalent residues to Trp25 and Lys26 of DTX-K, which may offer a hydrophobic surface for binding to the turret of K_v_1.1 subunits [14]. 

## 6. Molecular Diversity

As shown in this review, there are several protease inhibitors from venomous animals isolated and characterized from their venoms, body extracts or skin secretion so far. In addition, many of them were identified from transcriptomic approaches and their activity as protease inhibitors is still uncertain. 

In sea anemones, most of these polypeptides present the Kunitz-type serine protease inhibitor motif, with three conserved disulfide bridges (with exception of Inhibitor 4, from *Rhodactis rhodostoma*, which has only two disulfide bridges [31]), and thus are structurally homologous to the bovine pancreatic trypsin inhibitor (BPTI). Only two sea anemone polypeptides were shown to present distinct structural motifs to date—the cysteine inhibitor equistatin, with three thyroglobulin type-1 domains [43], and the non-classical Kazal-type elastase inhibitor (AEI) [30].

**Figure 2:**
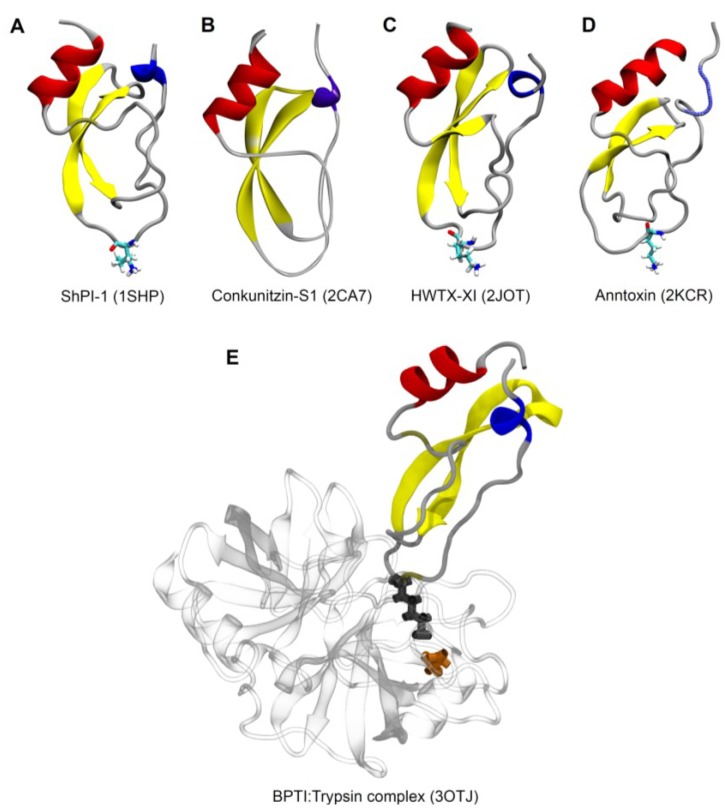
Tridimensional structures of polypeptides from venomous animals with the Kunitz-type inhibitor motif (**A**–**D**) and bovine pancreatic trypsin inhibitor (BPTI): trypsin complex (**E**). (**A**) ShPI-1 (PDB code 1SHP) from the sea anemone *Stoichactis helianthus*; (**B**) conkunitzin-S1 (PDB code 2CA7) from the cone snail *Conus striatus*; (**C**) HWTX-XI (PDB code 2JOT) from the spider *Ornithoctonus huwena*; (**D**) anntoxin (PDB code 2KCR) from the frog *Hyla annectans*; (**E**) BPTI:trypsin complex (PDB code 3OTJ). The structures are shown as ribbon diagrams, where α-helixes are indicated by red color, β-strands by yellow, 3_10_-helix by dark blue; and disordered structure by silver. In detail, Lys residue at P1 position (cyan at **A**, **C** and **D**). In BPTI:trypsin complex, interacting amino acid residues are shown in dark grey for BPTI (Lys15 residue at P1 position) and in orange for trypsin (Asp189 at S1 position). **A**, **C**, **D** and **E** were obtained with the software VMD (visual molecular dynamics) [185], whereas **B** was obtained with the software Discovery Studio 3.5 (Accelrys, Inc., San Diego, CA, USA).

The tridimensional structure of BPTI determined by both crystallography and NMR reveals an α/β/α structural motif [19,20,21]. BPTI contains a hydrophobic core and three disulfide bridges (C_I_–C_VI_, C_II_–C_IV_, C_III_–C_V_), and its structure is characterized by a 3_10_-helix at its *N*-terminal (residues 3 to 7), a β-hairpin of residues 18 to 35, an antiparallel β-strand involving residue 45 in contact to residue 21, and an α-helix formed by residues 47–56 at the *C*-terminal [19]. Structural-function relationship analysis of BPTI-enzyme complex has shown that a solvent exposed loop formed by residues 8 to 19 is highly complementary to the enzyme active site (S1 pocket), wherein the P1 residue (Lys15 in BPTI) deeply penetrates to interact with the S1 binding pocket of the protease. Trypsin, which has Asp189 as its S1 site, is particularly suited to interact with the basic side chains of Lys15 from BPTI [175,176] (Figure 2E).

Regarding the serine superfamily of PIs, three major classes are designated as trypsin-like (positively charged residues Lys/Arg preferred at P1), elastase-like (small hydrophobic residues Ala/Val at P1) or chymotrypsin-like (large hydrophobic residues Phe/Trp/Tyr/Leu/Val at P1) [12,84,177]. The P1 site residues Met, Asn and His for chymotrypsin binding were also reported [50,59,118,177,178]. A catalytic triad of residues (usually Ser, His and Asp) on the enzymes’ pocket is responsible for amide bond hydrolysis. Analysis of various mutants of BPTI and other trypsin inhibitors revealed that the interaction inhibitor:trypsin is almost independent of the nature of the basic residue at P1 position, with no significant changes in the association energies with trypsin after the mutation K15R in BPTI [179]. By contrary, inhibition for kallikrein favors Arg over Lys [180,181], while plasmin inhibition is enhanced by Lys at P1 site [182]. The P1′ site of venom trypsin/chymotrypsin inhibitors is occupied by a hydrophobic amino acid residue (Ala, Gly and Phe), being the Ala residue the most commonly used (Figure 1).

The solution structure of ShPI-1, a Kunitz-type protease inhibitor purified from the sea anemone *Stoichactis helianthus*, was determined by NMR spectroscopy [36]. Despite low sequence similarity between the two polypeptides (~35%), ShPI-1 (Figure 2A) has an almost identical molecular architecture to BPTI, with a 3_10_-helix involving residues 1 to 5, a twisted β-harpin of residues 16 to 33, a single-residue antiparallel β strand of residue 43 and an α-helix at the *C*-terminal (residues 45 to 54) [36]. Moreover, the crystallographic structure of ShPI-1 in complex with bovine pancreatic trypsin was revealed (PDB ID: 3M7Q) [39]. The overall structure of this complex is highly similar to the homologous complexes with BTPI (PDB ID: 2FTL and 3OTJ) [183], which is characterized by the lowest dissociation constant known so far for an inhibitor-protease interaction (*K*_i_ of 6 × 10^–14^ M) [184]. Around 40% of the total interactions at the interface rShPI-1A:trypsin are formed by Lys13 at P1 position (Figure 1, Figure 2A), whose side chain interacts with Asp189 at the trypsin catalytic pocket, similarly to BPTI [175]. Additional contributions to the stability of the complex ShPI-1:trypsin are indicated by residues Arg11 (P3 site) and Ile32. The side chain of Arg11 points directly into a pocket on the enzyme surface (S3 site), establishing additional H-bonds at the complex in comparison to BPTI, whose proline residue at this position does not deeply enter the S3 pocket due to its cyclic nature [178,183]. The *in silico* Arg11Ala mutation in ShPI-1 led to a 10-fold increase in the theoretical *K*_i_ value against trypsin. 

The sea anemone protease inhibitors kalicludines (AsKC1 to AsKC3) and ShPI-2, from *Anemonia sulcata* and *Stoichactis helianthus*, respectively, along with some Kunitz-type PIs from snakes, scorpions and spiders (Figure 1) also present Arg residue at the P3 position. Sea anemone kalicludines contain the residues Arg15 and Ala16 at P1 and P1′ positions, respectively, which are important for good trypsin binding. However, the presence of Pro19—equivalent to Pro21 in dendrotoxin, which is suggested to cause unfavorable feature for trypsin binding [153]—may explain the weaker inhibitory activities of kalicludines in comparison to BPTI, which has an Ile residue at the equivalent position [27]. 

The molecular diversity of Kunitz-type inhibitor homologs in *Heteractis crispa* was examined by using PCR-based cloning techniques, revealing that the *H. crispa* polypeptides are encoded by a multigene superfamily, which was divided into four distinct groups according to the *N*-terminal amino acid residues (GS-, RG-, GG-, and GN-) [15]. According to the nature of P1 residue, 33 deduced mature GS-polypeptides were categorized into three groups: group I (with Lys at P1 site), group II (Thr at P1), and group III (Arg at P1). Most of transcripts were placed in group I (66.1%), 24% generated group II, and 9.9% was in group III. Phylogenetic analysis suggested that the GS-polypeptide genes, as well as those from other sea anemone Kunitz-type inhibitors, have evolved from a common ancestor. The ancestral polypeptide more likely contains Arg as the P1 residue and Gly in position 38, as these important residues are shared by most of sea anemone Kunitz-type inhibitors (Figure 1) and members of group III [15].

The P1 site is occupied by a Thr residue, instead of Lys/Arg, in the sea anemone polypeptides InhVJ, Jn-IV and APHC1-APHC3 (Figure 1). Because the serine protease inhibitor InhVJ, from the sea anemone *Heteractis crispa*, is homologous to ShPI-1 (87% identity), its spatial structure could be model using the structure of ShPI-1 as a template, and the model was used to predict the structure of the complex InhVJ:trypsin [47]. Docking analysis showed a smaller number (three) of H-bond formed by the P1 Thr residue of InhVJ with the trypsin active site in comparison to those of ShPI-1 (five) and of BPTI (seven), which correlate well with the decreasing *K*_i_ values of their complexes. Electrostatic interactions and H-bonds of Glu45 at the weak contact site with protease Lys60 and Tyr39 were also important to stabilize the InhVJ:trypsin complex [47]. The conformational stability of InhVJ was analyzed by circular dichroism (CD) spectroscopy [186]. It was shown to be highly stable to changes in temperature and pH. Even after 70 °C, which is the point of the thermal conformational transition of the polypeptide, InhVJ still retained 80% of the inhibitory activity. Significant changes were observed on the level of tertiary structure of the molecule in the pH range 11.0–13.0, which may be explained by the ionization of tyrosine residues. InhVJ is conformationally stable at a low pH value (2.0). Quenching experiments showed that two tyrosine residues are completely accessible for the quencher, whereas the third residue is partially accessible [186].

From cone snail, only few PIs have been studied. Although sharing similar size and tridimensional structure to the Kunitz-type inhibitors, conkunitzin-S1 (Figure 2B) has only two disulfide bridges [105]. The solution structure of conkunitzin-S1 consists of a 3_10_-helix of residues 6–8, a contorted β-hairpin of residues 20–36, and an α-helix in the *C*-terminus (residues 50–56). The C_I_–C_IV_ pair connects the two helices, and the *C*-terminal helix is connected to the β-sheet by the disulfide bridge between C_II_ and C_III_ [187]. To evaluate the importance of the third disulfide bond to the K^+^-channel blocker activity, a double cysteine mutant (Conk-S1^CC^) with the additional cysteine bridge between the positions 16 and 40 was constructed. Interestingly, wild type and mutant Conk-S1 displayed the same inhibitory activity on *Shaker* channels [105]. Most of the K^+^-channel blockers from venomous animals have conserved residues, which are responsible for block of the ion conduction pore and promote a high affinity binding within the K^+^ channel pore vestibule, such as a lysine residue and an aromatic residue at 6.6 ± 1.0 Å from the α carbon of that lysine, respectively, a motif termed the “functional dyad” [188]. However, different interaction mechanisms have been described, among them, the “basic ring” (four or five non-identical basic residues that might stabilize the interaction with the channels) [189]. In the crystal structure of conkunitzin-S1, positive electrostatic potential is concentrated on one side of the molecule and there is apparently no dyad-like lysine-hydrophobic pair, suggesting that conkunitzin-S1 could interact with K^+^-channels through its arrangement of positively charged residues.

The scorpion mature peptide rLmKTT-1b (SdPI), from *Lychas mucronatus*, presents sequence similarity with Kunitz-type inhibitors. However, different from the typical Kunitz-type motif, rLmKTT-1b—along with the scorpion toxins rLmKTT-1a, rLmKTT-1c, and rBmKTT-1 [68]—possess a unique cysteine framework, where the normal C_II_–C_IV_ disulfide bridge is absent, such as conkunitizin-S1, but two additional cysteine residues at the *C*-terminus of the mature peptide might generate a new disulfide bridge. As a result, a distinct disulfide assignment may be generated (Figure 1) [16]. Also from scorpion venom PIs, recombinant BmKTT-2 was found to form four disulfide bridges (Table 1 and Figure 1), which is different from all known Kunitz-type animal toxins [68]. AvCI, from the spider *Araneus ventricosus* (Table 1) also presents the Kunitz-type motif and four disulfide bridges, and although a protease inhibitory activity was observed, this toxin was suggested to derive from the spider whole body, but not from venom [71].

These propositions were recently revealed with the solution structure of rLmKTT-1a [99]. NMR experiments of rLmKTT-1a showed a typical Kunitz-type fold, characterized by a *N*-terminal helix from Lys2 to Cys4, a double-stranded anti-parallel β-sheets from Phe17 to Asn23 and Lys28 to Tyr34, and a *C*-terminal helix from Asp49 to Ala55, where the disulfide link between Cys51 and Cys59 is presented. Although the sequence similarity is low between LmKTT-1a and classical Kunitz-type toxins, its structure resembles the fold of other inhibitors, such as conkunitzin-S1, dendrotoxin, HWTX-XI, and APEKTx1, suggesting that, despite of molecular diversity, these Kunitz-type peptides hold a structural conservation [99]. The mutant LmKTT-1a-C51A/C59A, with the same disulfide bridge pattern as those of cone snail conkunitzin-S1, and preserving a secondary structure similar to LmKTT-1a, presented a 5-fold lower trypsin inhibitory activity than the native LmKTT-1a, but the same K_v_1.3 channel blocking activity. 

The LmKTT-1a gene structure contains two introns in the mature peptide and differs from the genomic organization of the four families of scorpion KTxs, α-, β-, γ-, and κ-KTxs. Considering the structural, and functional features, and the genomic organization of scorpion Kunitz-type toxins, it has been proposed a new KTx subfamily to classify these scorpion peptides, the δ-KTx, where the seven members would be divided in three groups based on the disulfide bridge patterns [99].

Molecular dynamics simulation with a proposed rLmKTT-1b:trypsin complex model [16], based on the BPTI interaction with trypsin, showed the active site formed by Lys12, Gly13, Lys14 and Ala15 (Figure 1) inside the S1 pocket of trypsin. In the proximity of the active site there were also strong polar and nonpolar interactions among residues. Peptide Phe17 may also contribute to enhancing the rLmKTT-1b:trypsin interaction. The side chain of Lys14, the P1 residue of rLmKTT-1b, protrudes inside the S1 pocket and forms hydrogen bonds with trypsin residues Asp176 and Ser192. Therefore, it is suggested that besides its new disulfide bridge connection, rLmKTT-1b likely interacts with trypsin in a similar way to other Kunitz-type toxins [16]. 

Recently, four putative serine PIs belonging to the *Ascaris*-type peptides were identified in scorpion cDNA libraries [69]. Members of the *Ascaris*-type peptides’ family are characterized by four short β-strands arranged in two approximately perpendicular β-sheets and stabilized by five disulfide bridges [144]. 

The NMR solution structure of spider Kunitz-type toxin HWTX-XI (Figure 2C) [14] resembles the structures of BPTI and DTX-K [19,190]. In the *N*-terminal of HWTX-XI, from Thr3 to Arg5, there is a 3_10_-helix, and in the *C*-terminal, there is an α-helix from Gln45 to Cys52 and a triple-stranded anti-parallel β-sheet formed by Glu18–Asn23, Thr26–Ile31 and Lys41–Phe42. The two helixes are connected by the disulfide bond Cys4–Cys52, and the α-helix is connected to the β-sheet by the disulfide bond Cys27–Cys48. 

The studies with 18 mutants of HWTX-XI [14] showed that Lys14 is the main amino acid residue responsible for the trypsin inhibition function, since the mutation K14N reduced in approximately 10^5^-fold the inhibitory potency and K14A presented no trypsin binding activity at all. Thus, HWTX-XI counts with the presence of two separate and functionally independent sites—one for serine protease inhibition and another for potassium channel blockage (Figure 1), as discussed in the previous section.

By comparing the primary and tertiary structures of HWTX-XI and BPTI, the authors suggest some possible reasons for making HWTX-XI a stronger trypsin inhibitor than BPTI [14]. First, the replacement of Arg21 in BPTI by the smaller and non-charged residue Ser16 in HWTX-XI (Figure 1) increases the interaction of Lys14 with the negatively charged residue Asp189 at the bottom of the S1 pocket of trypsin. Second, the Phe17 of HWTX-XI has a larger side chain (phenyl group) than Ile22 of BPTI, and this may lead to a broader hydrophobic surface to interact with the hydrophobic pocket formed by Phe41 and Lys60 of trypsin. Third, the amino groups of the long hydrophobic side chain of Arg12 of HWTX-XI fit perfectly the positive pocket formed by the hydroxyls of Lys145, Ser146 and Gly148 of trypsin. These interactions may be strengthened by the potential hydrogen bonds between them [14].

As shown, most of snake venom serine protease inhibitors characterized to date also belong to the Kunitz-type motif, although some of them do not present inhibitory activity due to the absence of the key residues at P3–P3′ (Figure 1). 

Among the known protease inhibitors from venomous animals, PIs from Anurans display the major variety in structure length and number of cysteine residues. At least six different classes can be distinguished: long PIs, consisting of a basic single chain glycoprotein varying from 22 to 60 kDa; inhibitors with 56–61 amino acid residues and five disulfide bridges that, similar to scorpion SjAPI, are also members of the *Ascaris*-type peptides’ family; Kazal-type serine protease inhibitors varying from 52 to 78 amino acid residues and containing three disulfide bridges; Kunitz-type inhibitors also with three disulfide bridges and 57–62 residues long; long-chain inhibitors with 60 residues and only two disulfide bridges; and the two smallest protease inhibitors from venomous animals, with 17–18 amino acid residues and a single disulfide bridge. 

The mature OGTI, from the frog *Odorrana grahami*, is one of the smallest protease inhibitors ever found (17 amino acid residues) and contains a six-residue loop cross-linked by one disulfide bridge [86]. Circular dichroism spectroscopy analysis indicated that its conformational distribution is essentially random (94.8%). The importance of Lys13 to the inhibitory activity was confirmed by the mutation K13A, being the mutated peptide not active against trypsin [86]. 

The Bowman-Birk type trypsin inhibitor HV-BBI, from the frog *Huia versabilis*, is also one of the smallest PIs characterized from animals to date [87]. This 18-amino acid residue trypsin inhibitor contains a disulfide loop between Cys5 and Cys15. Substitution of Lys8 at the presumed P1 position in the conserved canonical BBI motif, -TKSIPP-, by Arg resulted in 3-fold decrease in trypsin inhibitory activity (*K*_i_ of ~57 nM), whereas replacement by the aromatic residue Phe resulted in complete ineffectiveness. Both the Lys-P1 and Arg-P1 variants had no inhibitory activity against chymotrypsin even at a concentration of 100 µM. In contrast, the Phe-P1 variant presented a modest chymotrypsin inhibitory activity (*K*_i_ of ~389 nM). Thus, the Lys residue that occupies the P1 position seems to be optimal for potency of action against trypsin [87].

The amphibian peptide anntoxin, from *Hyla annectans*, is similar to conkunitzin-S1, having the same four cysteine framework and spacing [85]. The NMR structure of the recombinant peptide (Figure 2D), which presented protease inhibitory activity equal to the native peptide, was also similar to those of conkunitzin-S1 [105]. Its structure consists of a twisted β-hairpin (Thr20–Phe34), an α-helix (Leu49–Ala59), and a short 3_10_-helix (Tyr4–Cys6) in the *N*-terminus. Their two disulfide bridges are formed by Cys6–Cys56 and Cys31–Cys52. In anntoxin, the conserved trypsin inhibitor domain and conserved interactive sites were found to be the residues ^14^KGSGST^20^ [85]. 

Two other Kunitz-type inhibitors characterized from Anurans, KTPI (*K*unitz-*t*ype *p*rotease *i*nhibitor) and KSCI, were isolated from *Dyscophus guineti* and *Kassina senegalensis*, respectively [17,84], and their P1 residues (Figure 1) are in complete agreement with their trypsin (Arg16 in KTPI) and chymotrypsin (Phe17 in KSCI) inhibitory activities. 

Among the few protease inhibitors functionally characterized from wasp and bees until now, three of them (bicolin, Bi-KTI and Bt-KTI) share the three conserved disulfide bridges of Kunitz/BPTI-type inhibitors (Figure 1). Besides having an unusual P1 residue (Gln15), bicolin, from the venom of the wasp *Vespa bicolor*, still shows trypsin and thrombin inhibitory activity [88].

## 7. Therapeutic Potential

Besides being one of the most studied small globular proteins used as tool for protein 3D structure, protein/protein interaction, and molecular recognition studies, the clinical use of BPTI has been advised in cardiac surgery and orthotopic liver transplantation. BPTI has a very high specificity for plasmin, and exerts a significant reduction in hemorrhagic complications, blood-transfusion requirements, and the inflammatory status associated to extra-corporeal circulation [191,192]. Indeed, patients treated with BPTI submitted to repeated cardiac surgery, valve replacement and coronary-artery bypass grafting, presented up to 80 percent less blood loss and up to 60 percent lower transfusion requirements than control groups [193,194]. The potential use of textilin-1, a serine protease inhibitor isolated from snake venom, is proposed to reduce blood loss during cardiopulmonary surgery [7,122,195].

As broadly known and emphasized in the present review, proteases are essential for most physiological processes and, because of that, protease inhibitors have a wide therapeutic potential on cancer and on CNS, cardiovascular, inflammatory and neurodegenerative diseases, including Alzheimer's disease (reviewed in [11]), as well as on viral and parasitic infections associated to overexpressed or unregulated enzyme activity. More than 50 human diseases have been associated to specific mutations in protease genes [196].

Cysteine protease inhibitors have been proposed as antimalarial drugs, because of the critical role for these proteases in hemoglobin hydrolysis at the trophozoite stage of malaria parasite [197]. These proteases also take part in many human physiological pathways such as antigen processing and prohormone activation, as in some diseases including arthritis, Alzheimer’s disease, and cancer-cell invasion, reinforcing the importance of finding new cysteine protease inhibitors.

According to practice guidelines published by the American Association for the Study of Liver Diseases (AASLD) in 2011, the best treatment for genotype 1 chronic hepatitis C virus (HCV) infection is a triple therapy that consists of ribavirin and pegylated interferon-α together with a serine protease inhibitor (telaprevir or boceprevir) [198]. In the Acquired Immunodeficiency Syndrome (AIDS), the HIV protease has become an important therapeutic target leading to the development of several protease inhibitors [199,200], and their use as therapeutic drugs has been associated with a drastic reduction in AIDS morbidity and mortality, although the benefits have been compromised by viral mutation and drug-resistance [201].

## 8. Conclusions

Several protease inhibitors have been isolated from venomous animals, mainly sea anemones, snakes and Anurans. Nevertheless, the characterization of PIs from scorpions, spiders and Hymenopterans has attracted increasing interest from the scientific community, whereas those from cone snails remain less studied. Among the serine protease inhibitors, these polypeptides usually have distinct specificity for different proteases, such as trypsin, chymotrypsin, elastase, plasmin, and others. Because most of toxins from venomous animals are characterized as Kunitz-type serine protease inhibitors and present similar tridimensional structural motifs, their specificity towards serine proteases is mainly associated with P1 amino acid. However, besides the amino acid residues surrounding the reactive site, the residues present in the weak contact loop are also important for the different interactions with various serine proteases.

In addition to the protease inhibitory activity, some of these polypeptides are also blockers of potassium channels, being the site of interaction with these channels different from and independent of that for the protease binding. Evolutionary features suggest that new potassium channel blockers or dual-function neurotoxins have been originated from ancient molecules. 

Because of the increasing diversity of known Kunitz-type polypeptides, primary sequence comparisons, along with studies comprising site-directed mutagenesis and conformational analysis, could help better understand which amino acid residues are essential for protease inhibition and also for the dual-function. By analyzing the sequences of BmKTT-1, BmKTT-2 and BmKTT-3 (Figure 1), for example, it is possible to propose that the higher potency of BmKTT-1 on K_v_1.3 channels in relation to these other toxins is because only BmKTT-1 possesses the dyad residues Lys2 and Leu5 equivalent to those in dendrotoxins. Conversely, LmKTT-1a also possesses these dyad residues, but displays little blocking activity. One possible explanation could be the lack of the second cluster of key residues in the equivalent β-turn region of dendrotoxins. The sea anemone toxin APEKTx1, in turn, is a potent blocker of K_v_1.1 channels, and although not containing the corresponding Lys5 and Leu9 residues of α-DTX and DTX-I, it contains the residues Leu7 and Lys10, and also the second cluster of key residues. These two regions could be responsible for the higher potassium channel activity in relation to SHTX-3, which is a weak channel blocker. However, the sea anemone kalicludines, which present most of the key residues for K^+^ channel blocking activity, are weak inhibitors of K_v_1.2 channels. Thus, it would be interesting to test them on different subtypes of potassium channels, such as K_v_1.1 and K_v_1.3.

This sort of analysis could lead to the design of more potent protease inhibitors and dual-function polypeptides, and also to their strategically minimization in size, fashioning enhanced and low-priced drugs for diverse therapeutic and biotechnological applications. Moreover, comparative analysis could lead to a global evolutionary model that comprises all these PIs, which are undoubtedly provided with important physiological functions in the source organisms.
